# Supplementary figures and images for: Inferring Population Size History from Large Samples of Genome-Wide Molecular Data - An Approximate Bayesian Computation Approach
Source: PLoS Genet. 2016 Mar 4;12(3):e1005877. doi: 10.1371/journal.pgen.1005877 (PMC4778914; doi:10.1371/journal.pgen.1005877)

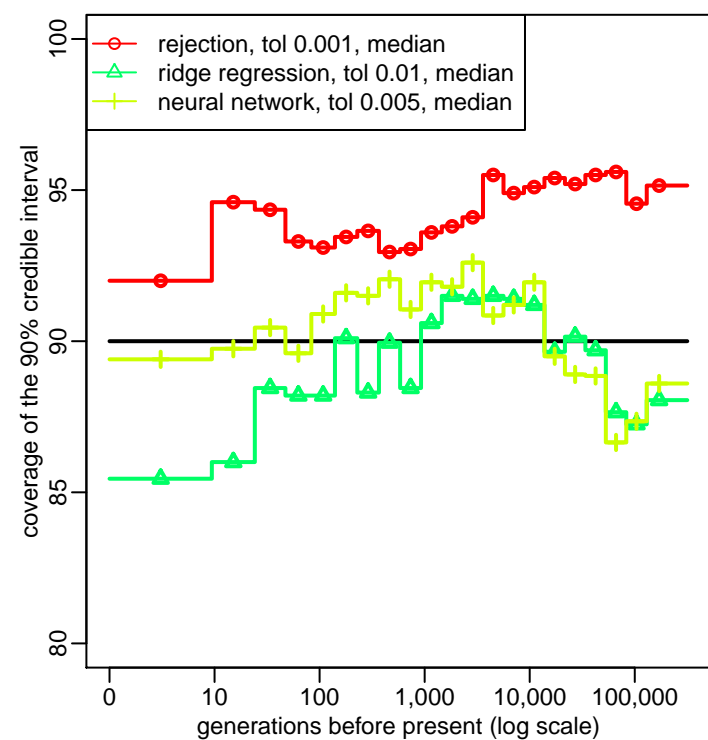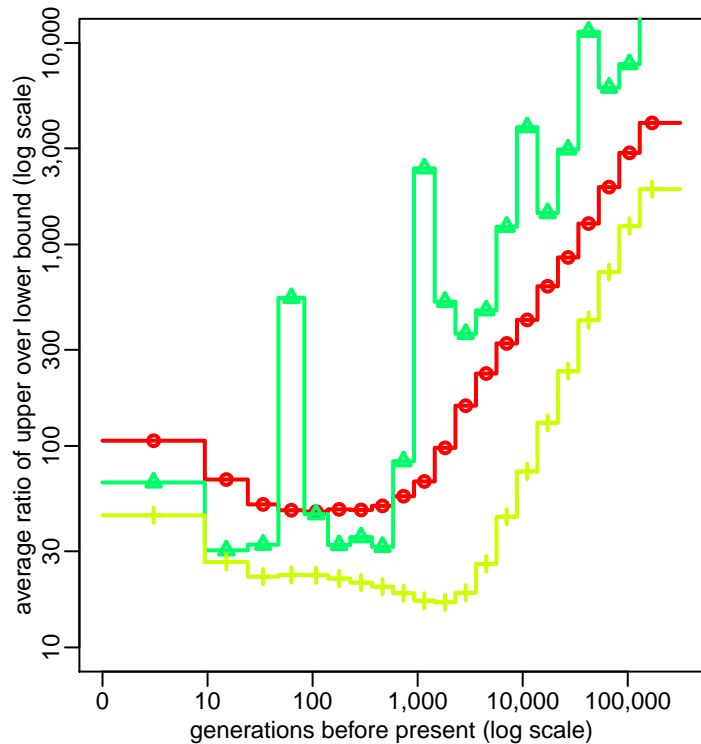

Supplement: S1 Fig — Empirical coverage (left) and width (right) of the 90% credible interval for the population size in each time window. The empirical coverage is the proportion of simulated histories for which the true population size was included in the 90% credible interval of the posterior distribution. If the posterior distribution was correctly estimated, this proportion should have been 90%, as shown by the black horizontal solid line. Parameter settings were the same as in Fig 1. (PDF) [file pgen.1005877.s001.pdf]

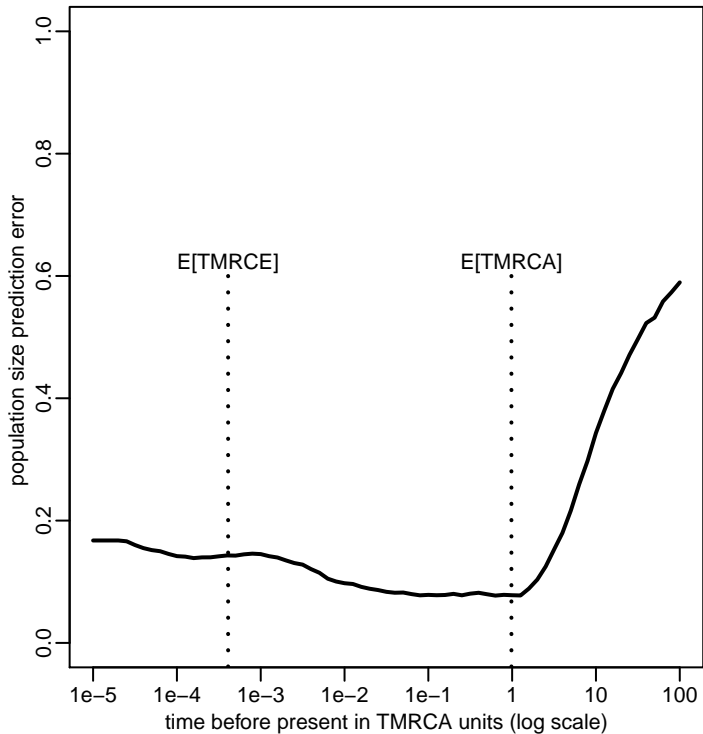

Supplement: S2 Fig — Prediction error for the estimated population size when time is measured in units of the expected time to the most recent common ancestor (TMRCA) of the sample. Prediction errors were evaluated from 2,000 random population size histories. Black vertical dotted lines indicate the expected time to the most recent coalescence event, E[TMRCE], and the expected TMRCA, E[TMRCA]. Summary statistics considered in the ABC analysis were (i) the AFS and (ii) the average zygotic LD for several distance bins. These statistics were computed from n = 25 diploid individuals, using all SNPs for AFS statistics and SNPs with a MAF above 20% for LD statistics. The posterior distribution of each parameter was obtained by neural network regression [32], with a tolerance rate of 0.005. Population size point estimates correspond to the median of the posterior distribution. (PDF) [file pgen.1005877.s002.pdf]

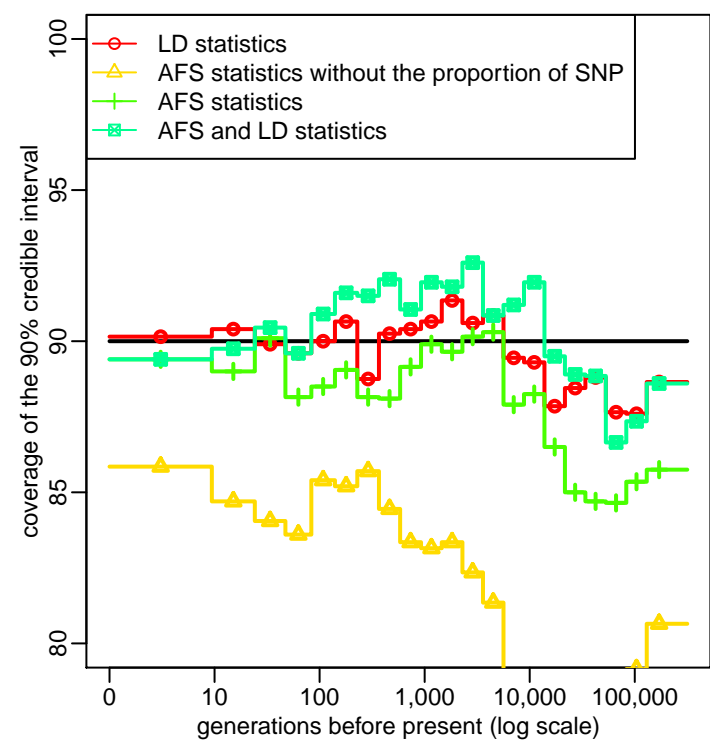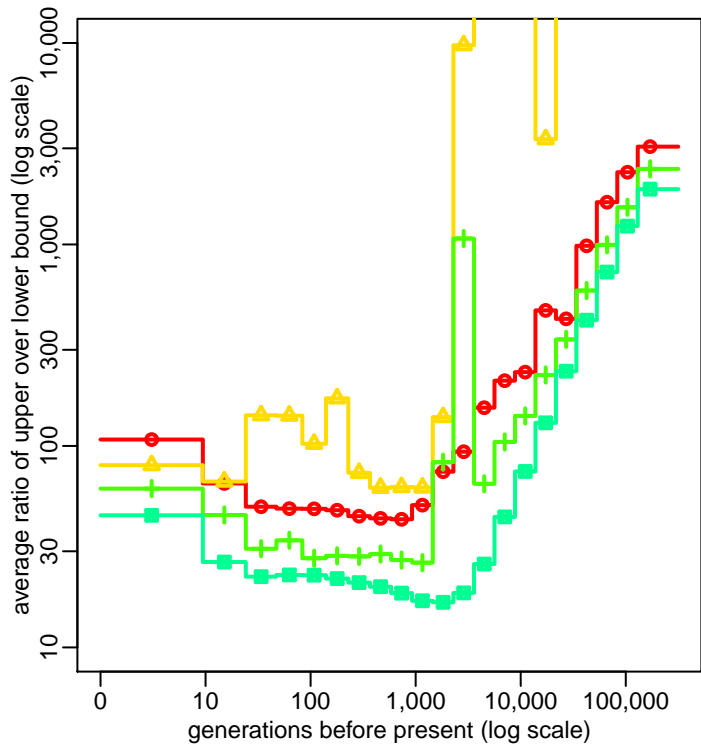

Supplement: S3 Fig — Empirical coverage (left) and width (right) of the 90% credible interval for the population size in each time window. Parameter settings were the same as in Fig 2. The very large credible intervals obtained on average with AFS statistics, in some time windows, are due to a retalively small number of PODs with extreme values. (PDF) [file pgen.1005877.s003.pdf]

population size prediction error

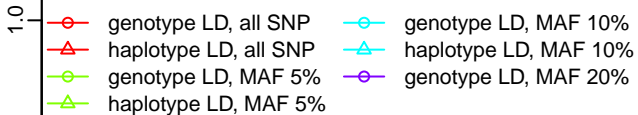

0

10

100

1,000

10,000

100,000

generations before present (log scale)

Supplement: S4 Fig — Prediction error for the estimated population size in each time window, evaluated from 2,000 random population size histories. Summary statistics considered in the ABC analysis were the average gametic LD (triangles) or the average zygotic LD (circles) for several distance bins. These statistics were computed from n = 25 diploid individuals, using different MAF thresholds. Other parameter settings were the same as in Fig 2. (PDF) [file pgen.1005877.s004.pdf]

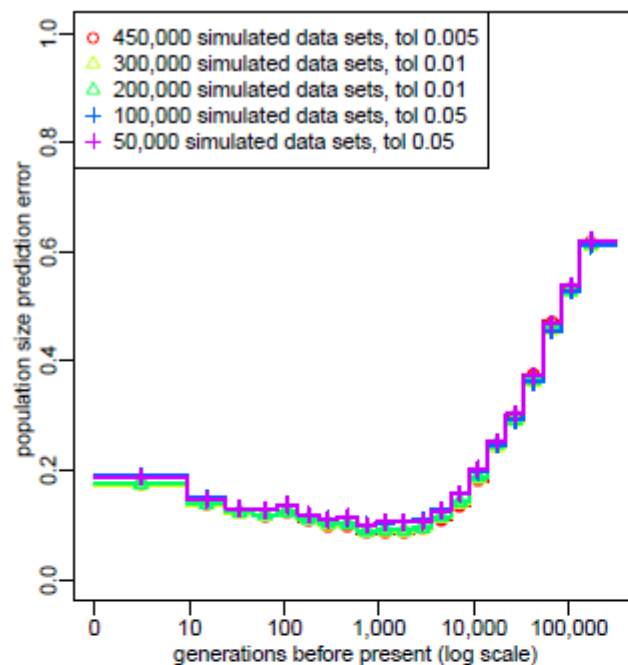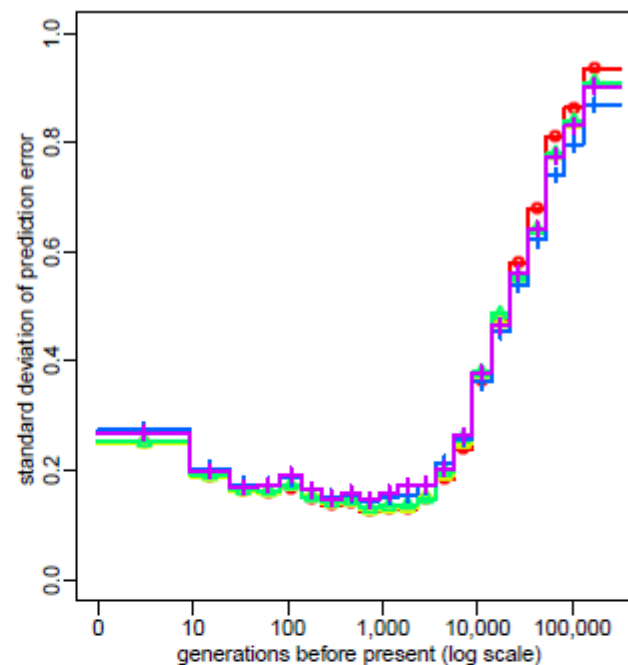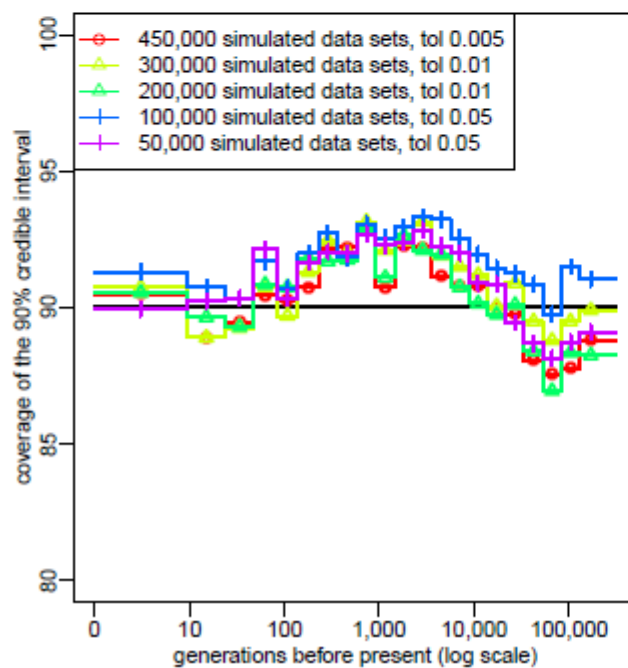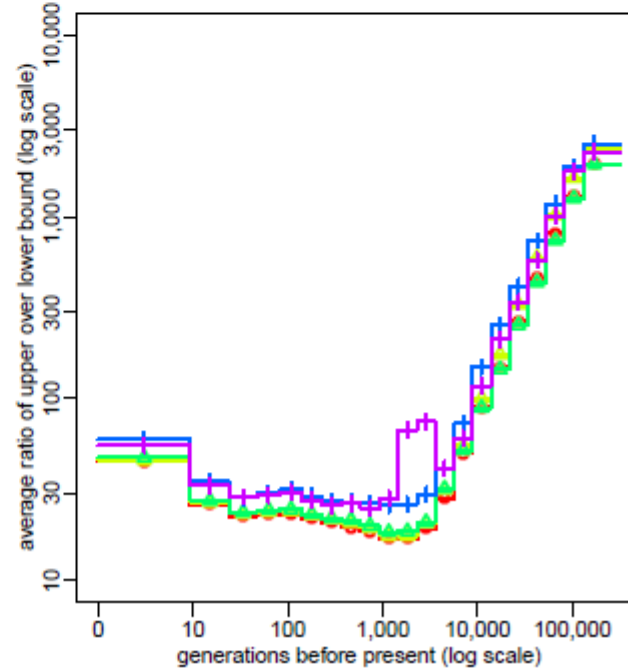

Supplement: S5 Fig — Top: Prediction error for the estimated population size in each time window (left) and standard deviation of this error (right). Bottom: Empirical coverage (left) and width (right) of the 90% credible interval for the population size in each time window. These quantiles were evaluated from 2,000 random population size histories. For each of these histories, one POD of n = 25 diploid genomes was simulated, where each genome consisted in 100 independent 2Mb-long segments. Population size history was estimated from this POD by ABC, for various numbers of simulated datasets (see the legend) with the same sample size (n = 25) and genome length (100 independent 2MB segments). Summary statistics considered in the ABC analysis were (i) the AFS and (ii) the average zygotic LD for several distance bins. AFS statistics were computed using all SNPs and LD statistics were computed using SNPs with a MAF above 20%. The posterior distribution of each parameter was obtained by neural network regression, with the tolerance rate leading to the smallest prediction error. Population size point estimates were obtained from the median of the posterior distribution. (PDF) [file pgen.1005877.s005.pdf]

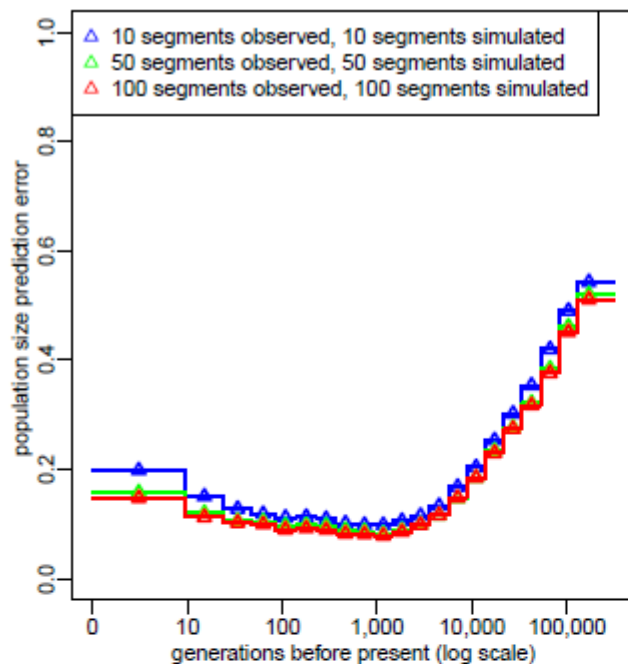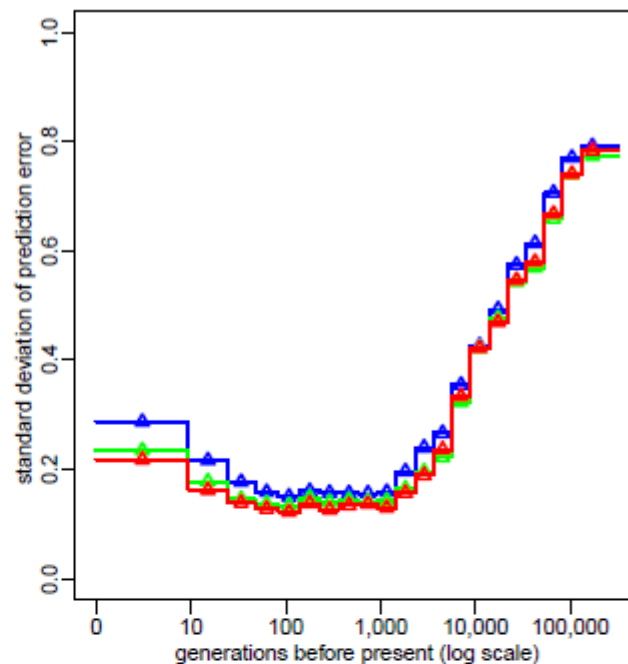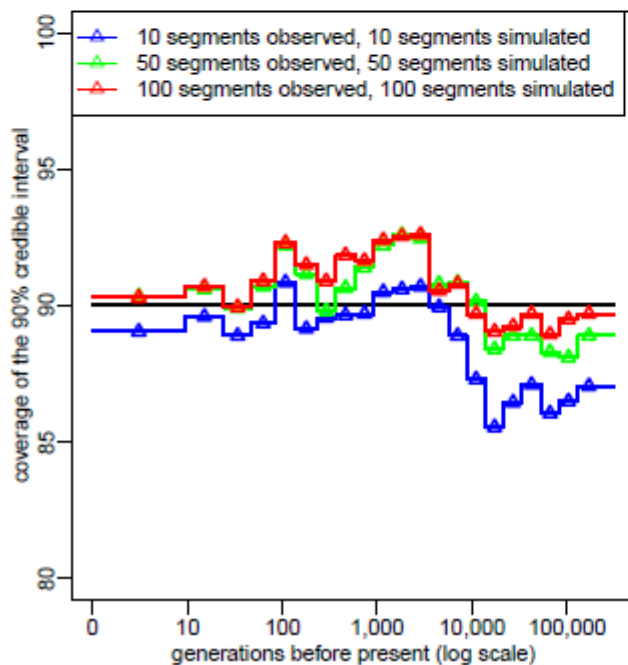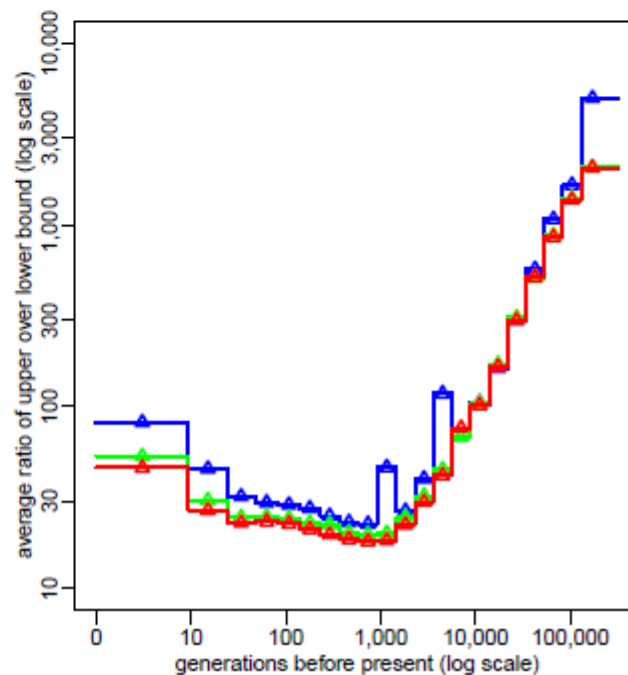

Supplement: S6 Fig — Top: Prediction error for the estimated population size in each time window (left) and standard deviation of this error (right). Bottom: Empirical coverage (left) and width (right) of the 90% credible interval for the population size in each time window. These quantiles were evaluated from 2,000 random population size histories. For each of these histories, one POD of n = 25 diploid genomes was simulated, where each genome consisted in 10, 50 or 100 independent 2Mb-long segments (see the legend). Population size history was estimated from this POD by ABC, using 450,000 simulated datasets with the same sample size (n = 25) and genome length. The posterior distribution of each parameter was obtained by neural network regression, with a tolerance rate of 0.005. All other settings are similar to S5 Fig. (PDF) [file pgen.1005877.s006.pdf]

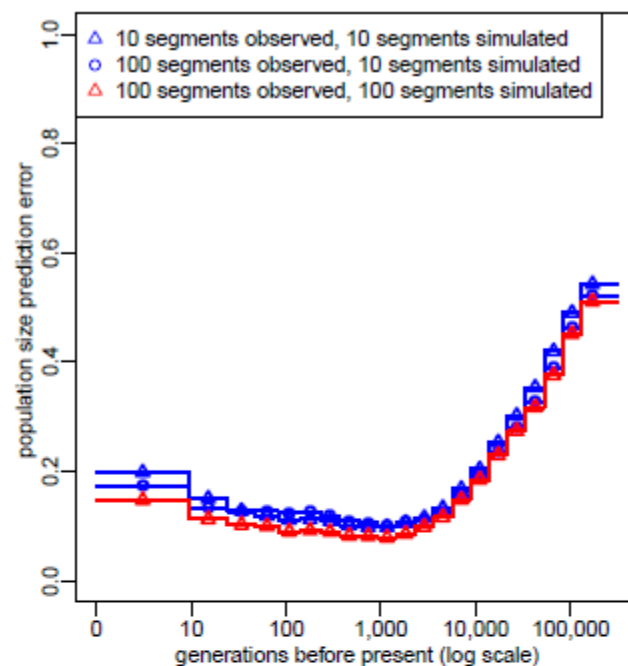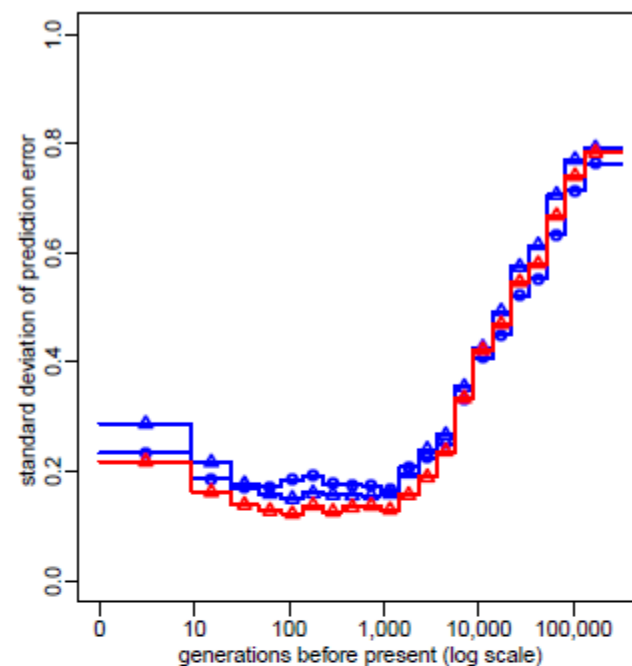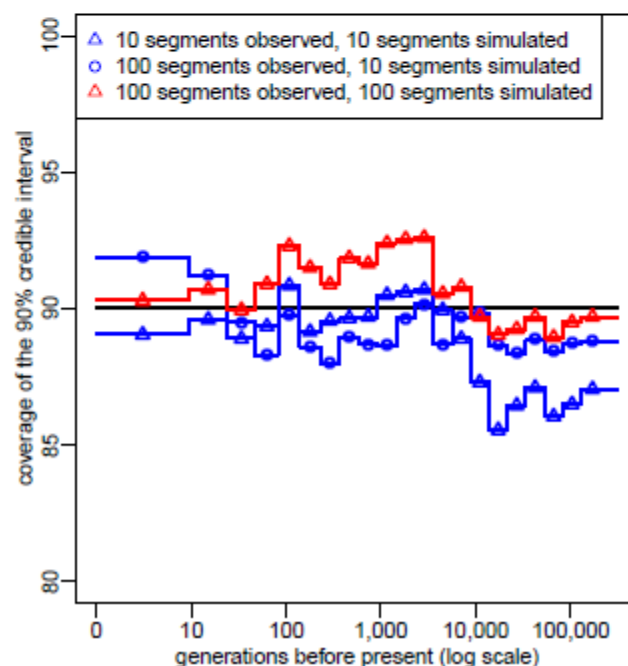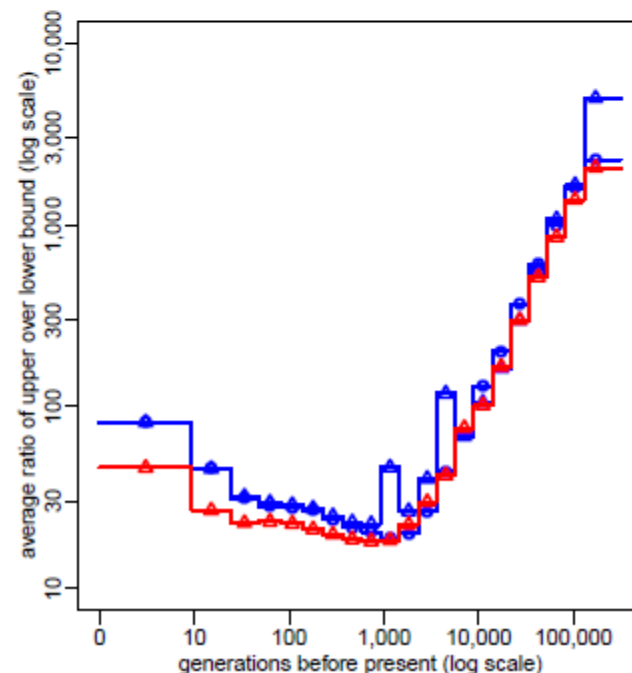

Supplement: S7 Fig — Top: Prediction error for the estimated population size in each time window (left) and standard deviation of this error (right). Bottom: Empirical coverage (left) and width (right) of the 90% credible interval for the population size in each time window. These quantiles were evaluated from 2,000 random population size histories. For each of these histories, one POD of n = 25 diploid genomes was simulated, where each genome consisted in 10 or 100 independent 2Mb-long segments (see the legend). Population size history was estimated from this POD by ABC, using 450,000 simulated datasets with the same sample size (n = 25) but a possibly different genome length (see the legend). The posterior distribution of each parameter was obtained by neural network regression, with a tolerance rate of 0.005. All other settings are similar to S5 Fig. (PDF) [file pgen.1005877.s007.pdf]

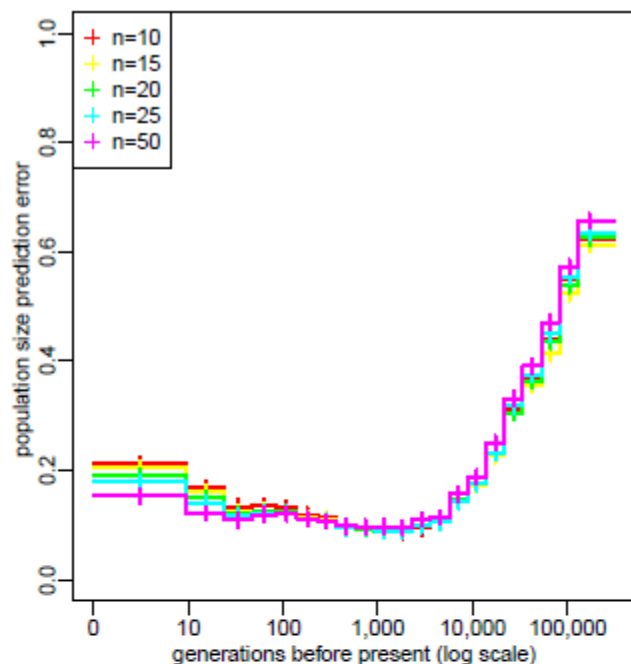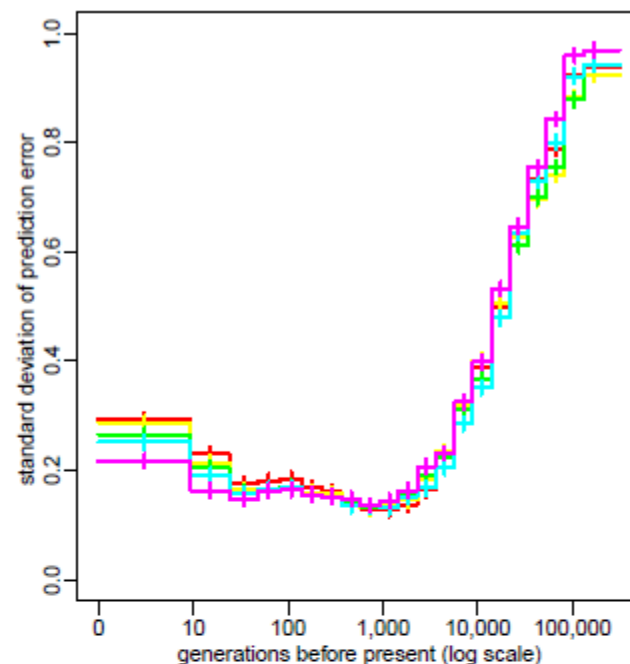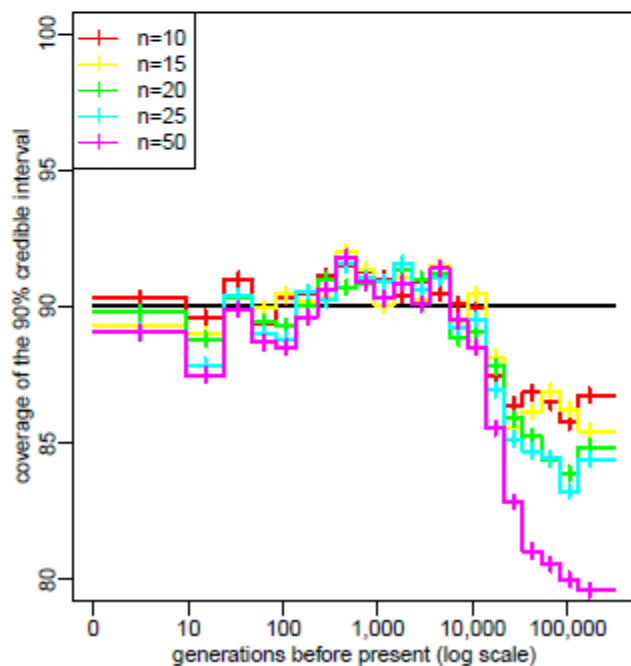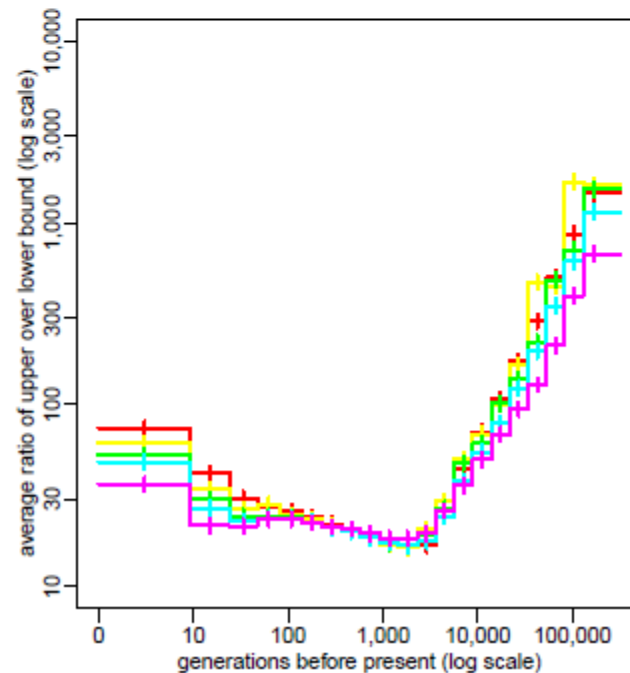

Supplement: S8 Fig — Top: Prediction error for the estimated population size in each time window (left) and standard deviation of this error (right). Bottom: Empirical coverage (left) and width (right) of the 90% credible interval for the population size in each time window. These quantiles were evaluated from 2,000 random population size histories. For each of these histories, one POD of n diploid genomes was simulated, for different values of n between 10 and 50 (see the legend). Each genome consisted in 100 independent 2Mb-long segments. Population size history was estimated from this POD by ABC, using 450,000 simulated datasets with the same sample size and genome length. All other settings are similar to S5 Fig. (PDF) [file pgen.1005877.s008.pdf]

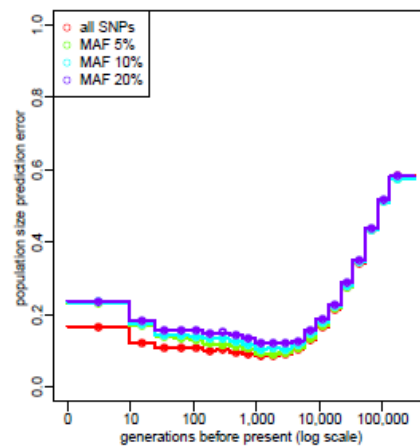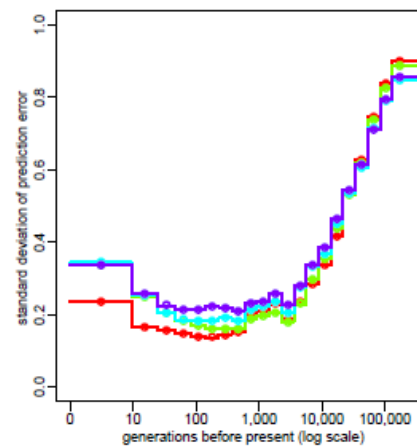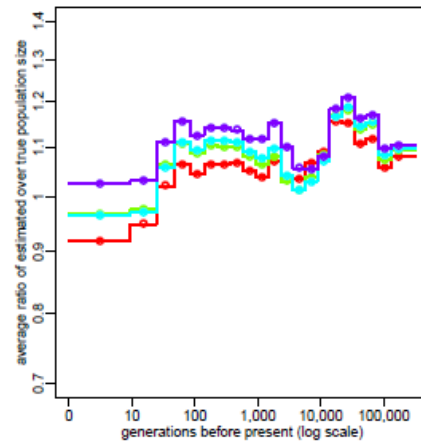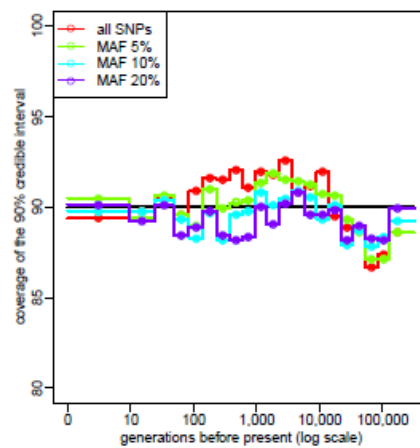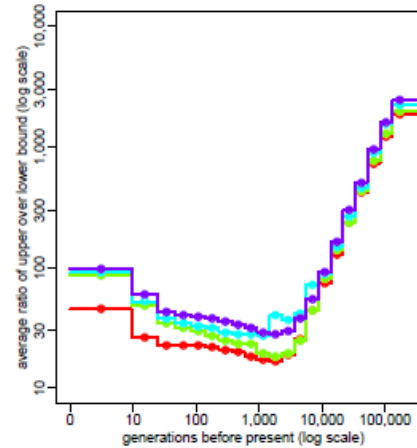

Supplement: S9 Fig — Top: Prediction error for the estimated population size in each time window (left) and standard deviation of this error (right). Middle: Bias for the estimated population size in each time window. Bottom: Empirical coverage (left) and width (right) of the 90% credible interval for the population size in each time window. These quantiles were evaluated from 2,000 random population size histories. For each of these histories, one POD of n = 25 diploid genomes was simulated, where each genome consisted in 100 independent 2Mb-long segments. Population size history was estimated from this POD by ABC, using 450,000 simulated datasets with the same sample size and genome length. Summary statistics considered in the ABC analysis were (i) the AFS and (ii) the average zygotic LD for several distance bins. AFS statistics were computed using different MAF thresholds, LD statistics were computed from SNPs with a MAF above 20%. The posterior distribution of each parameter was obtained by neural network regression, with a tolerance rate of 0.005. Population size point estimates were obtained from the median of the posterior distribution. (PDF) [file pgen.1005877.s009.pdf]

**small**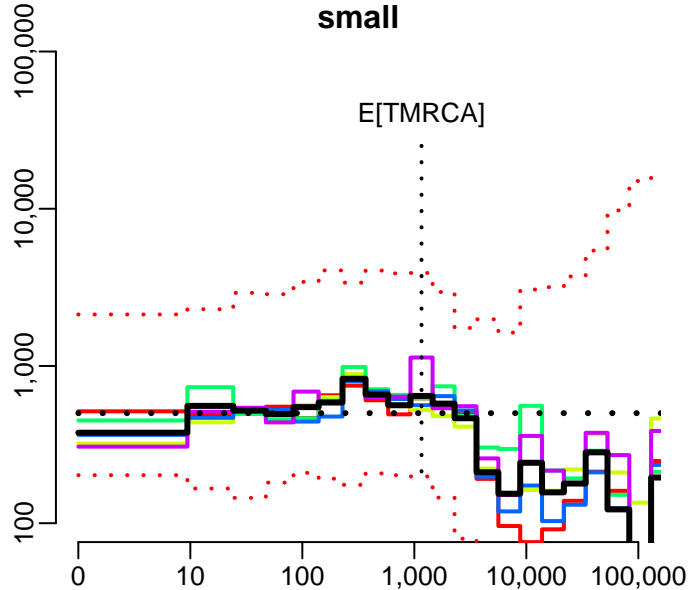**large**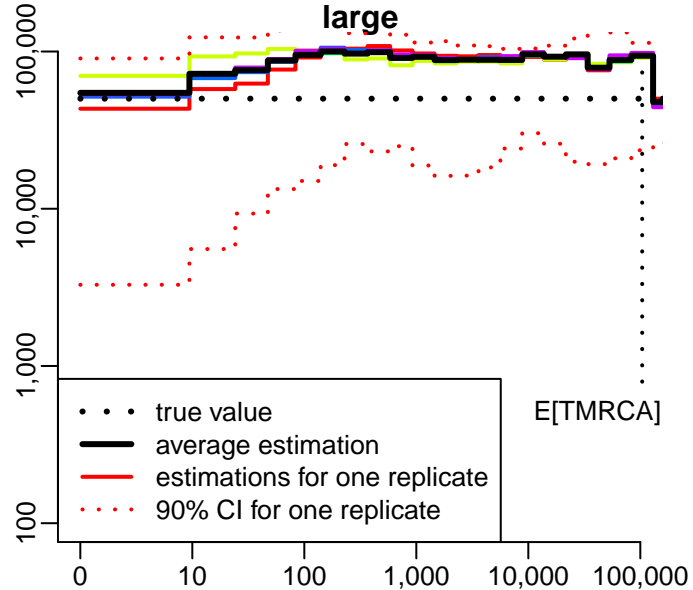**decline**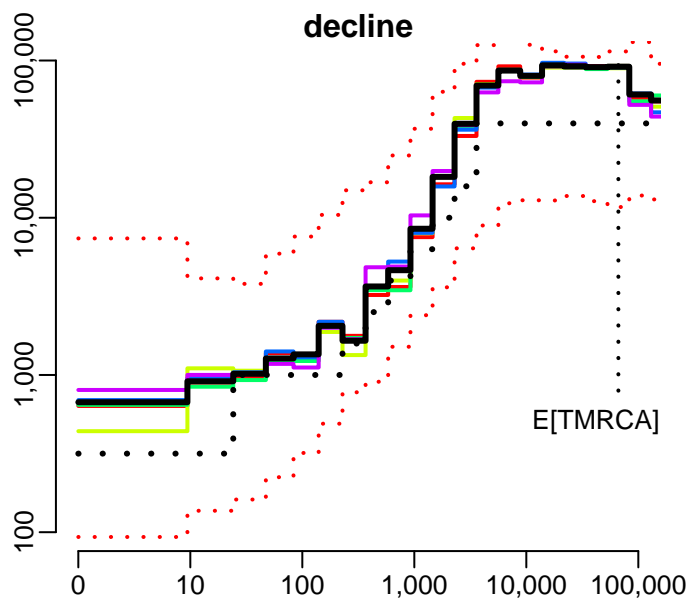**expansion**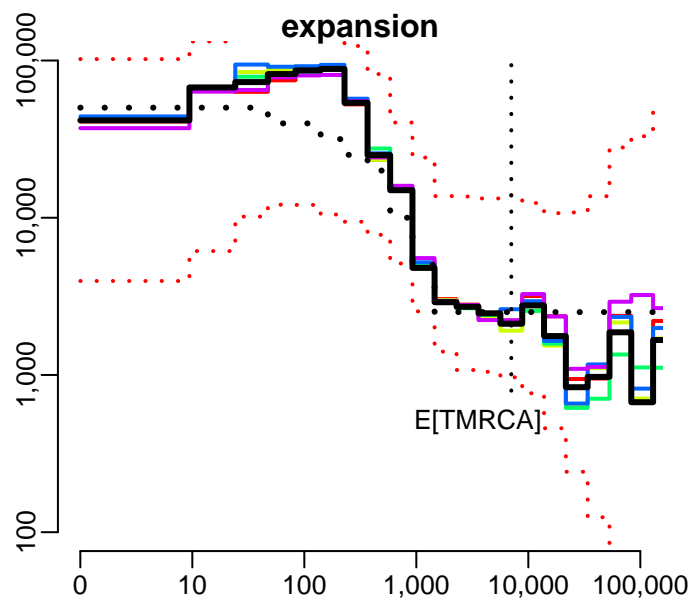**bottleneck1 old large**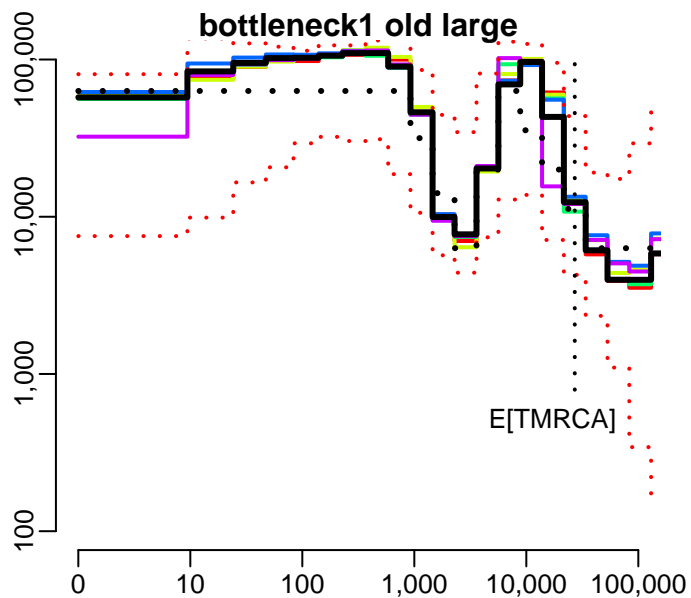**zigzag large**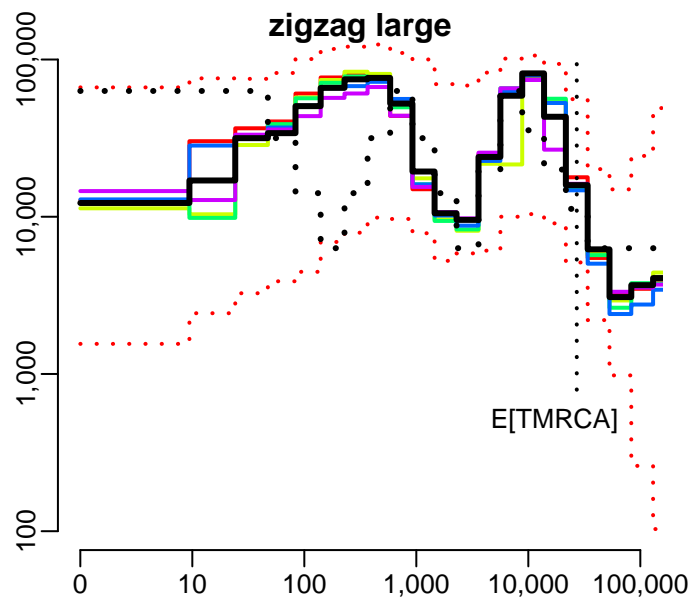

Supplement: S10 Fig — All settings are similar to Fig 3, except that population size point estimates were obtained from the mode of the posterior distribution. (PDF) [file pgen.1005877.s010.pdf]

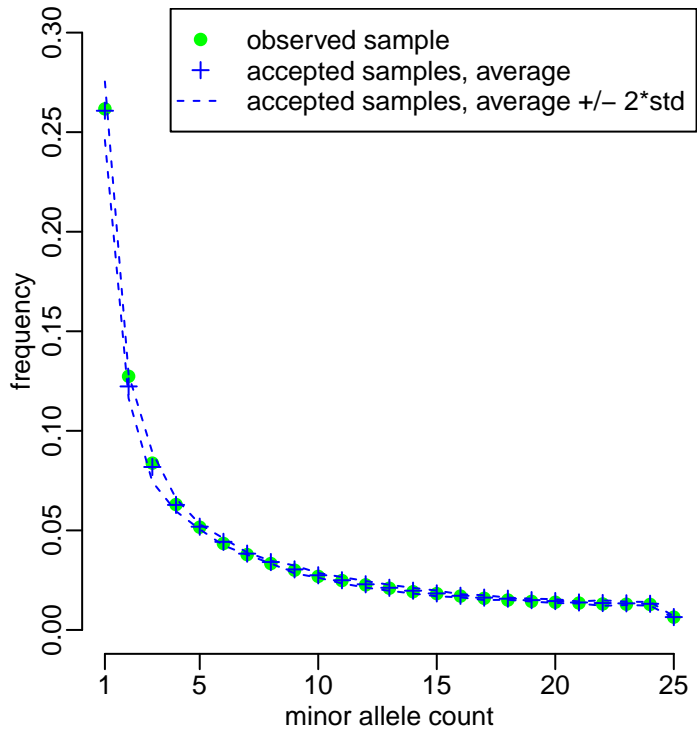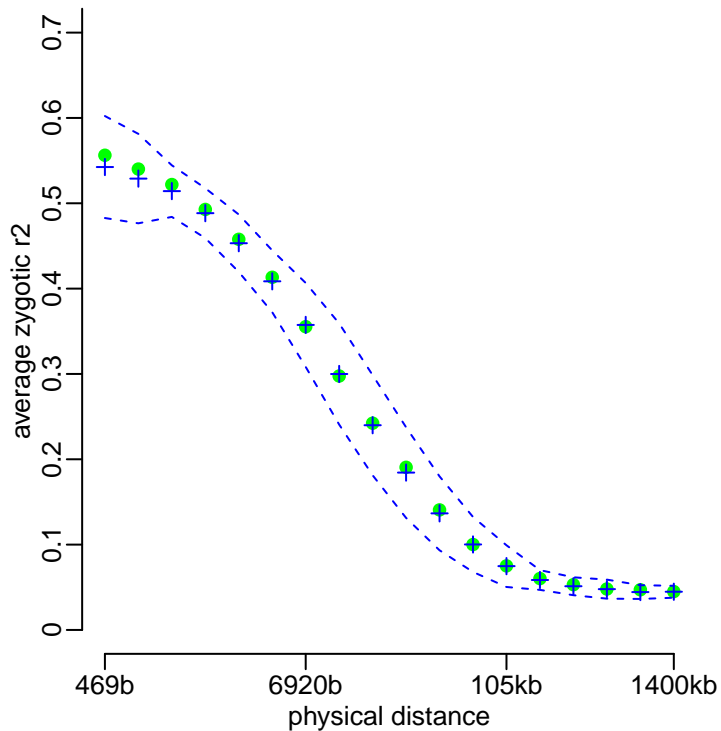

Supplement: S12 Fig — For one of the five PODs analyzed in this scenario, observed AFS (left) and LD (right) statistics are shown by green full circles. The average value of these statistics over the five best simulated data sets, i.e. the five simulated data sets leading to the smallest distance between observed and simulated statistics, are shown by blue crosses. The variation of these statistics over the five best simulated data sets is also indicated by blue dotted lines, which correspond to the average value plus (or minus) twice the standard deviation of each statistic. (PDF) [file pgen.1005877.s012.pdf]

**bottleneck2 recent large**

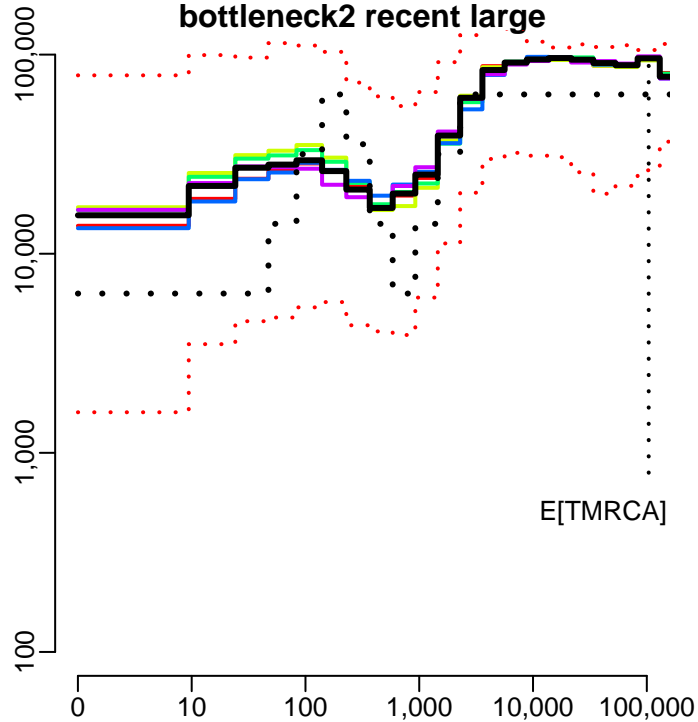

**bottleneck2 old large**

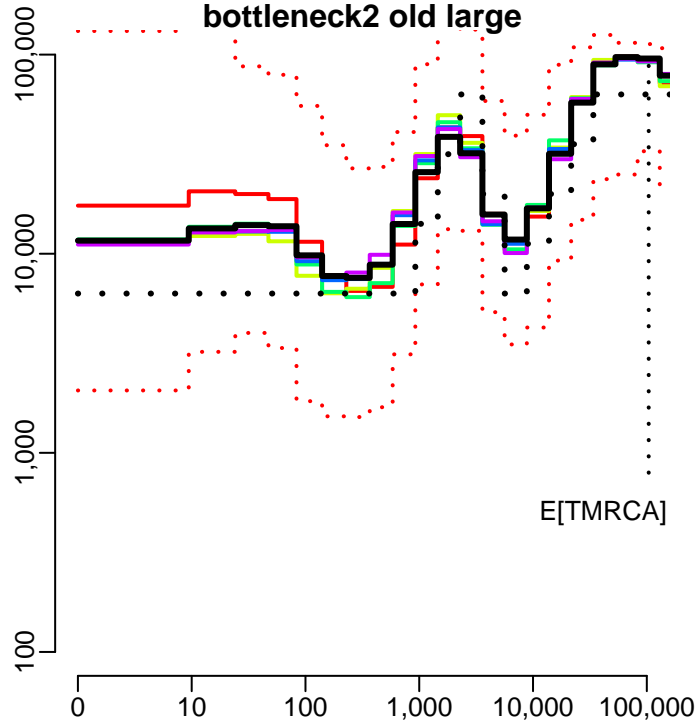

**bottleneck2 recent small**

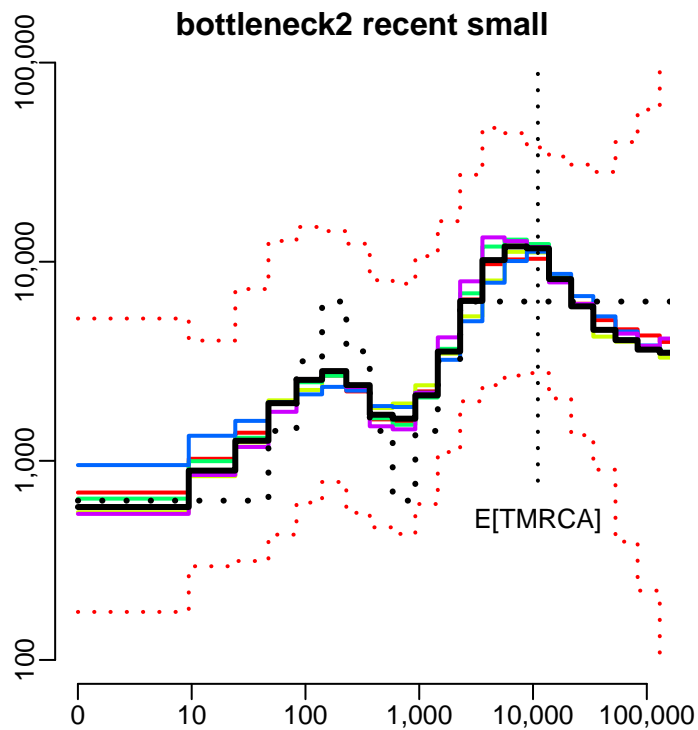

**bottleneck2 old small**

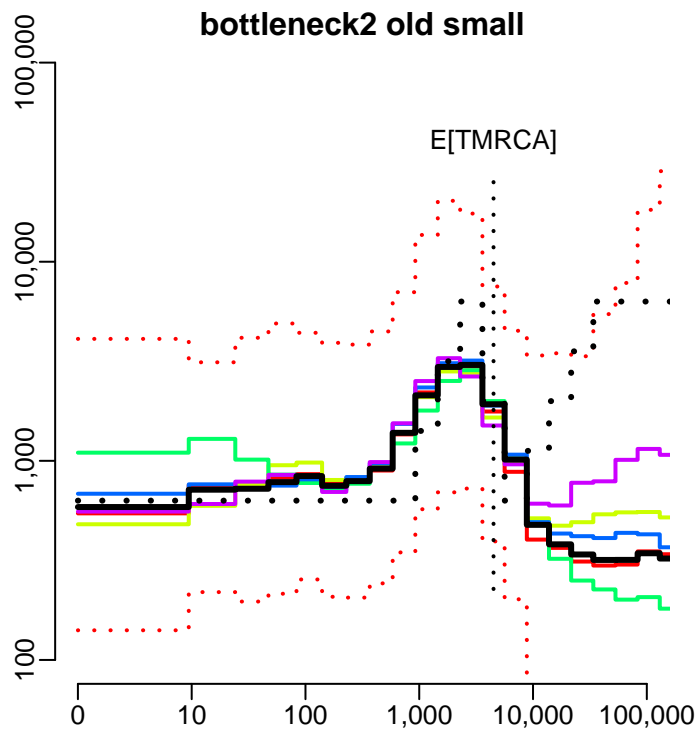

Supplement: S13 Fig — Population size varied between 60,000 and 6,000 individuals in the top panels, and between 6,000 and 600 individuals in the bottom panels. Population size changes occurred between 2,300 and 50 generations BP in the left panels, and between 34,000 and 900 generations BP in the right panels. All settings are similar to Fig 3. (PDF) [file pgen.1005877.s013.pdf]

**small**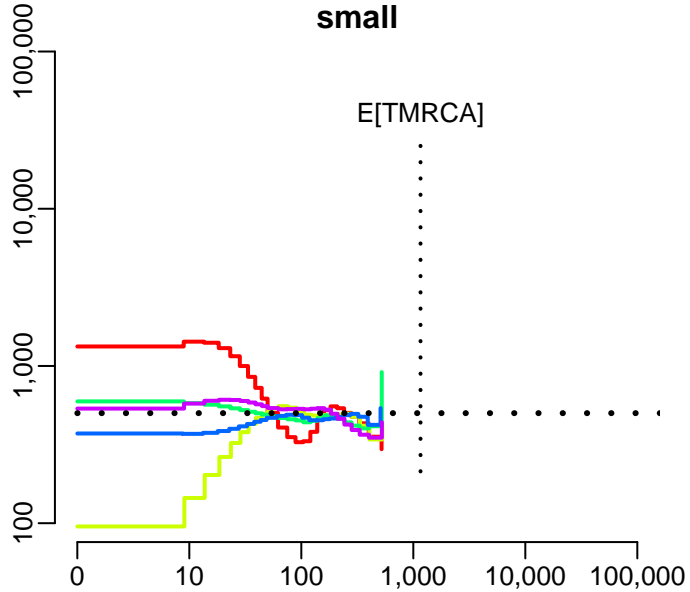**large**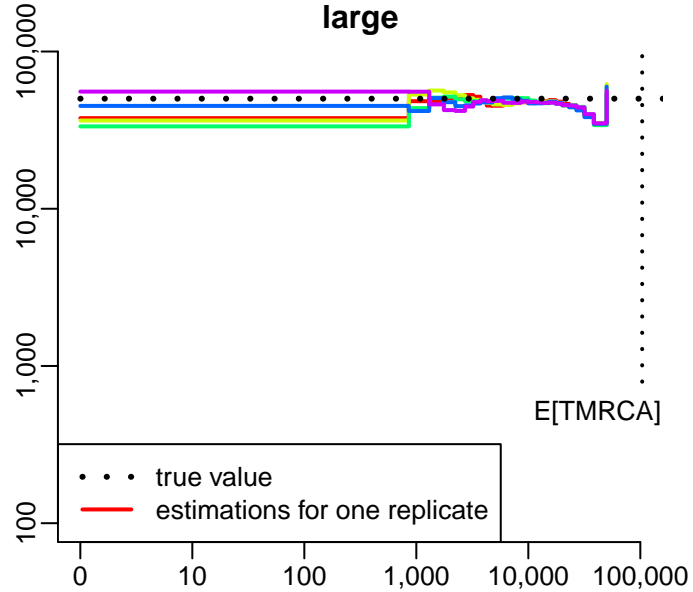**decline**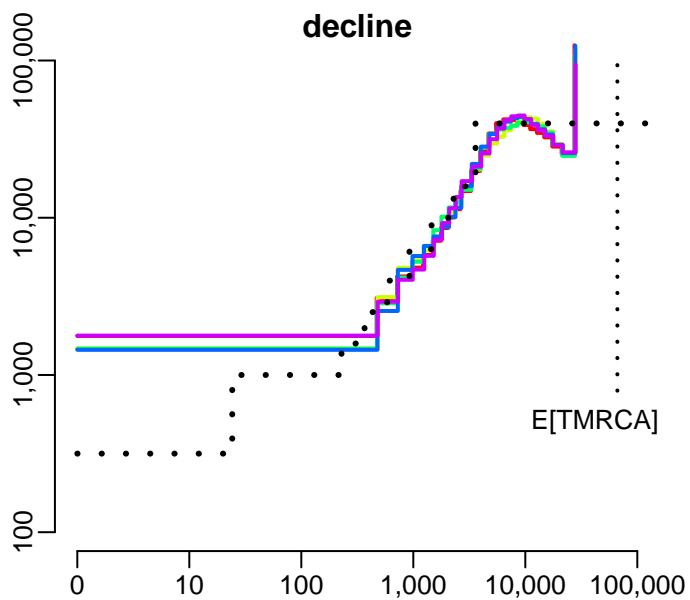**expansion**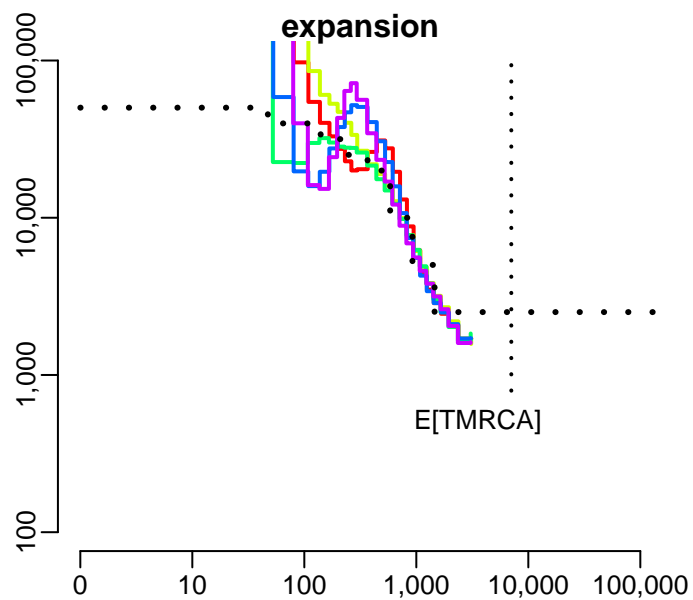**bottleneck1 old large**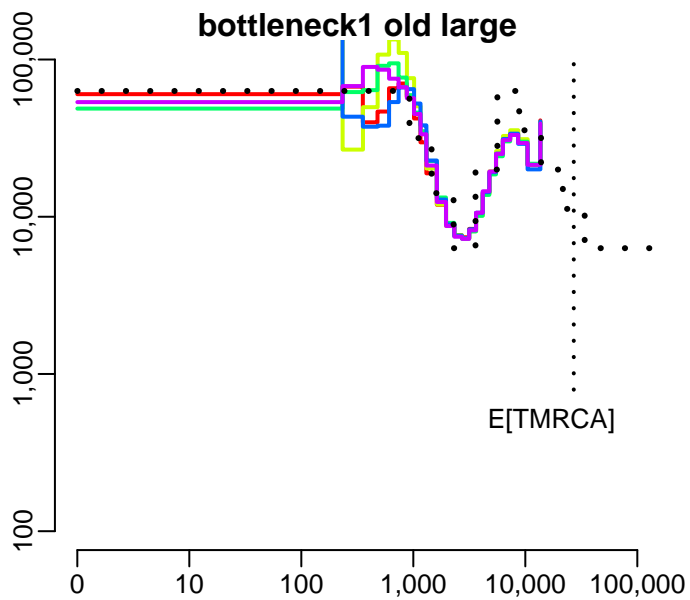**zigzag large**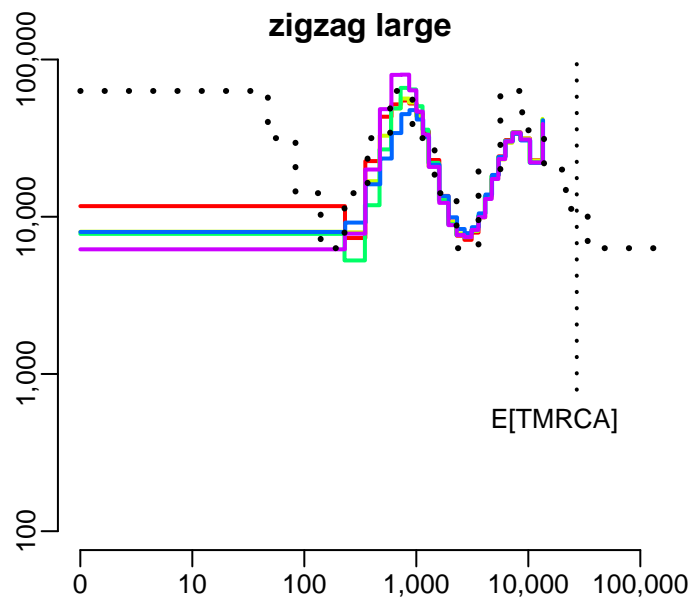

Supplement: S15 Fig — For each scenario, the five PODs considered for MSMC estimation were the same as in Fig 3. The expected TMRCA shown here is also the same as in Fig 3, it corresponds to samples of 50 haploid sequences. (PDF) [file pgen.1005877.s015.pdf]

**small**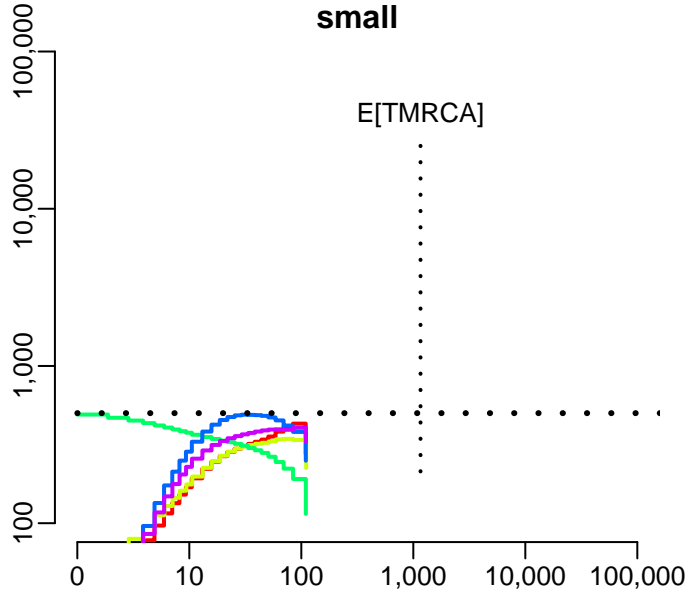**large**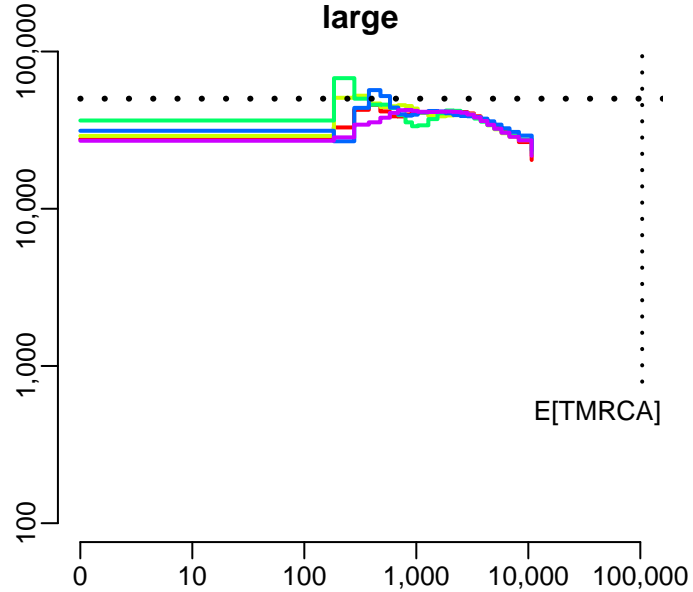**decline**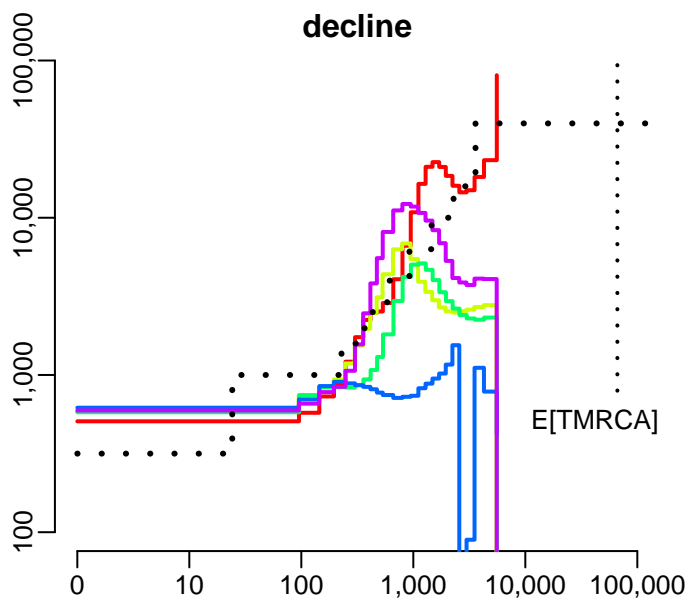**expansion**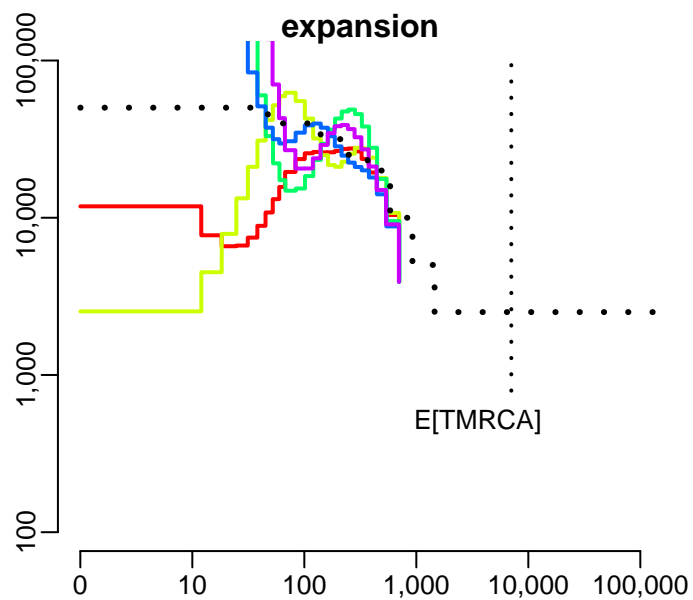**bottleneck1 old large**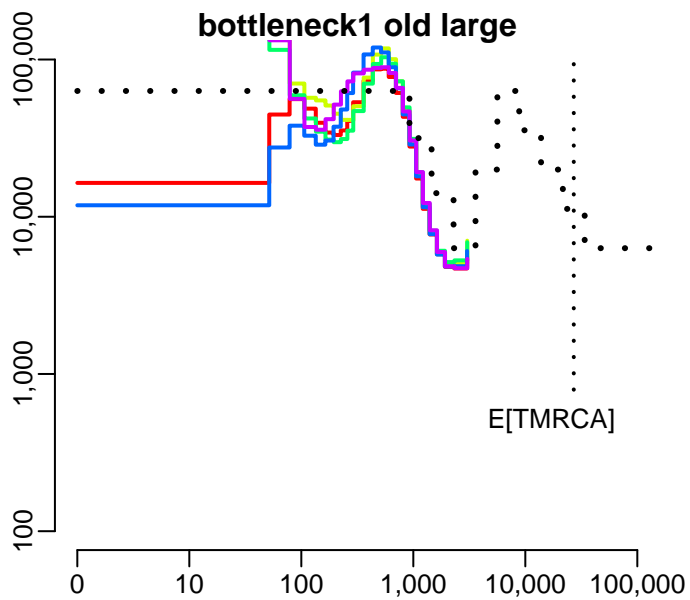**zigzag large**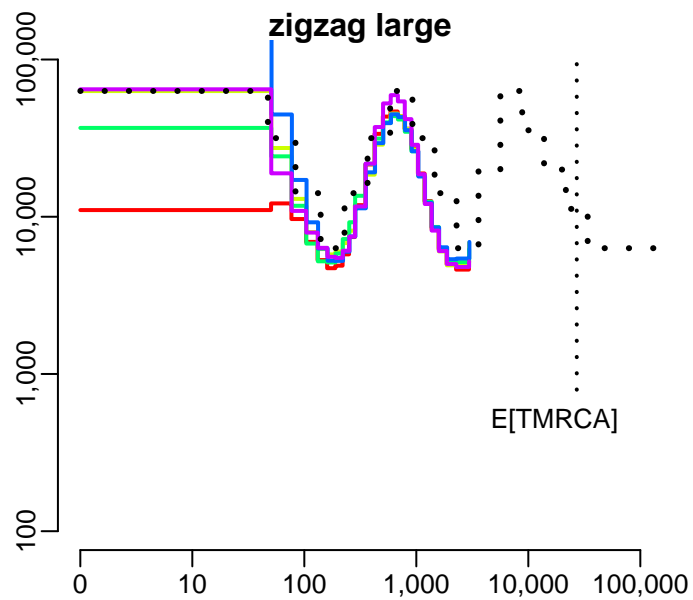

Supplement: S16 Fig — For each scenario, the five PODs considered for MSMC estimation were the same as in Fig 3. The expected TMRCA shown here is also the same as in Fig 3, it corresponds to samples of 50 haploid sequences. (PDF) [file pgen.1005877.s016.pdf]

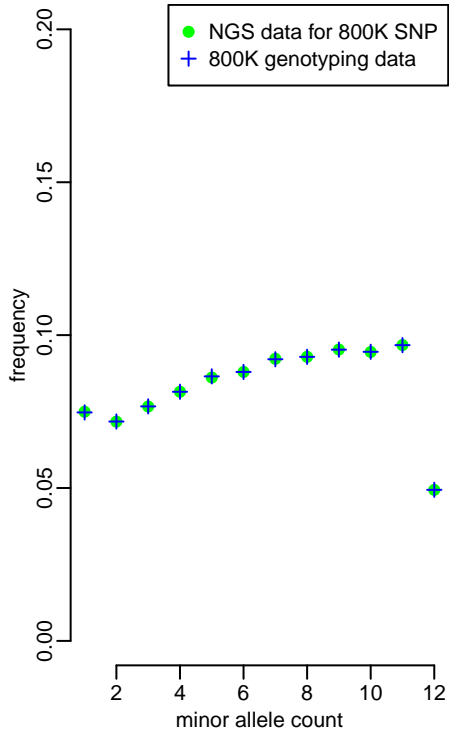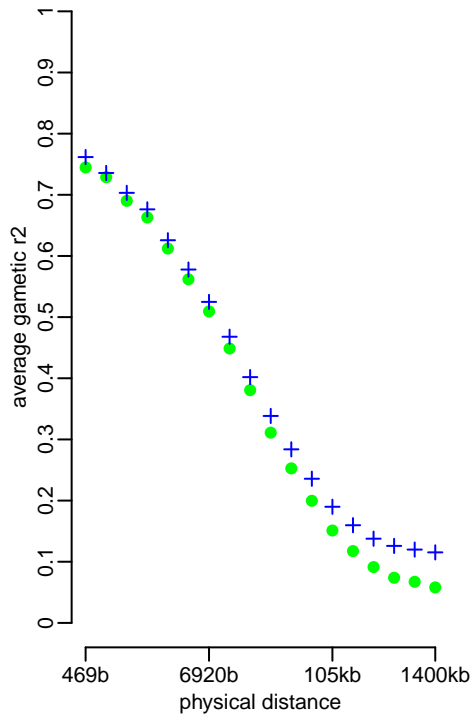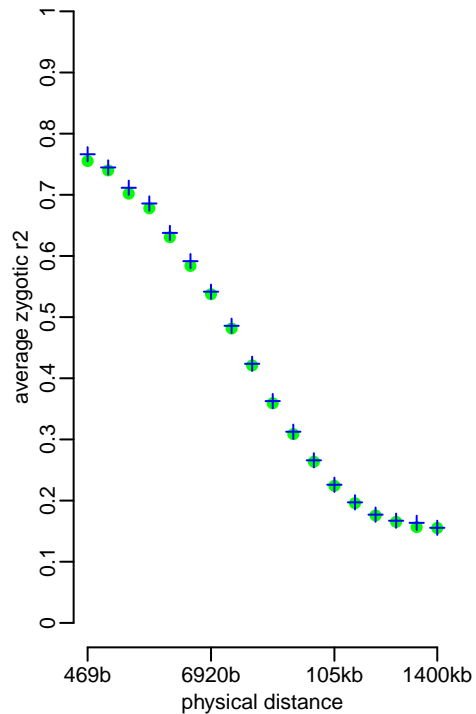

Supplement: S20 Fig — polymorphic site AFS, i.e. without the overall proportion of SNPs (left), average gametic LD (middle) and average zygotic LD (right). These statistics were computed from 12 Holstein animals for which both NGS data and genotyping data were available, using only SNP positions from the 800K chip (even for the NGS data statistics). No MAF threshold was used. (PDF) [file pgen.1005877.s020.pdf]

false positive rate

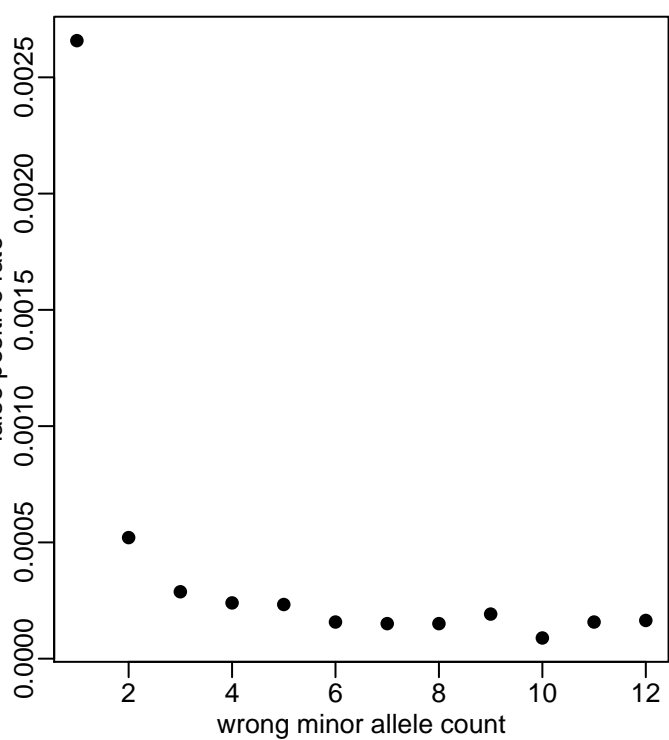

false negative rate

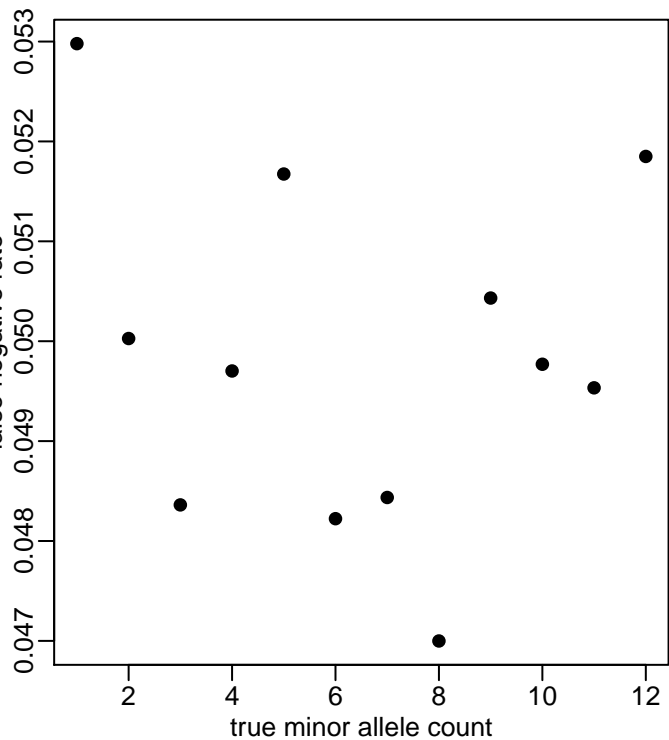

Supplement: S21 Fig — Error rates were computed from 12 Holstein animals for which both NGS data and genotyping data were available. False positive SNPs were positions that were found polymorphic in the NGS data but not in the 800K data. Their minor allele count in the NGS data was called the wrong minor allele count. False negative SNPs were positions that were found polymorphic in the 800K data but not in the NGS data. Their minor allele count in the 800K data was called the true minor allele count. (PDF) [file pgen.1005877.s021.pdf]

**small**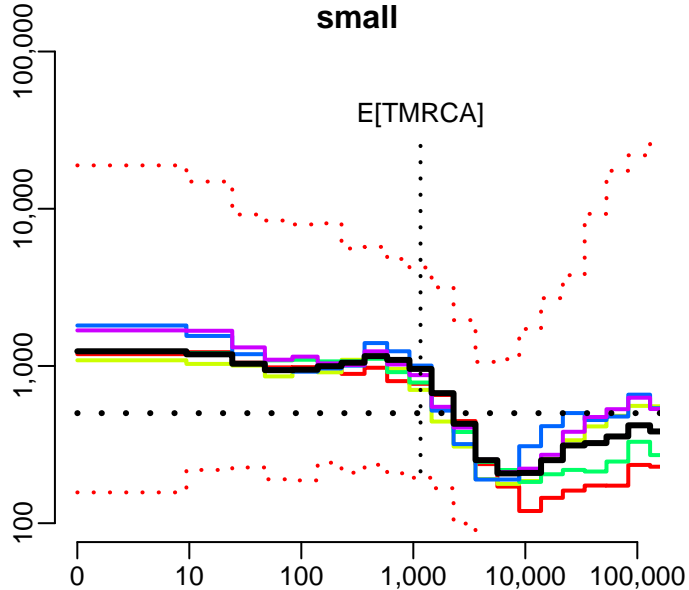**large**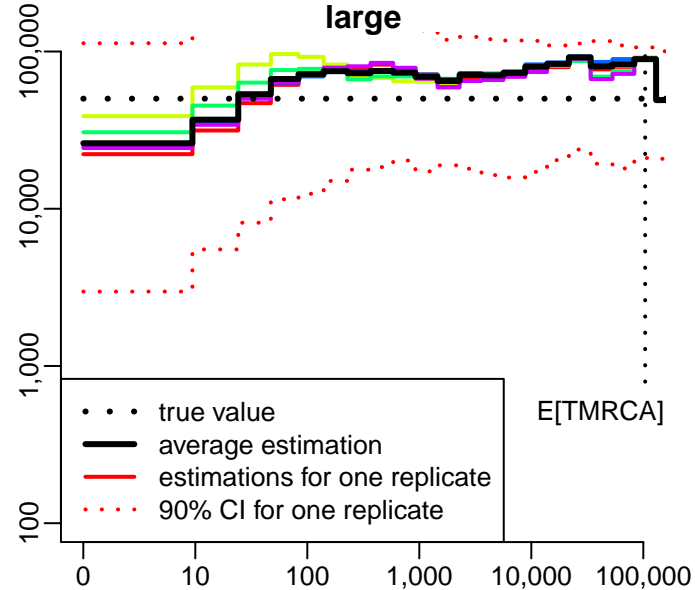**decline**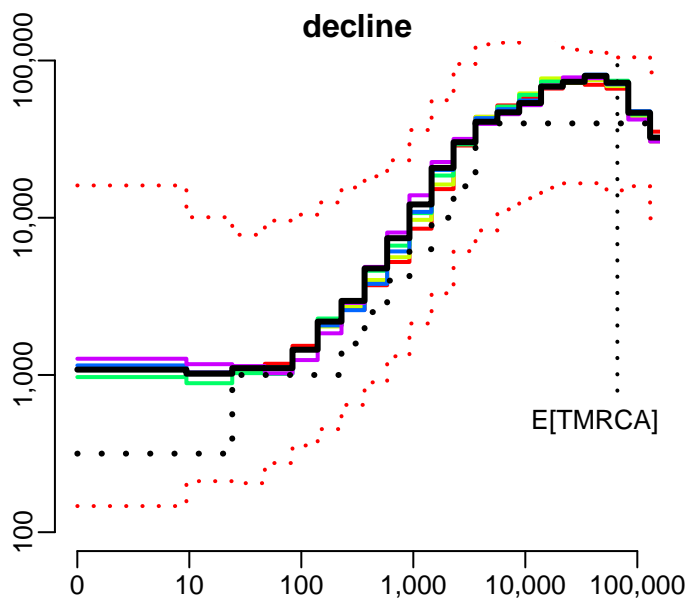**expansion**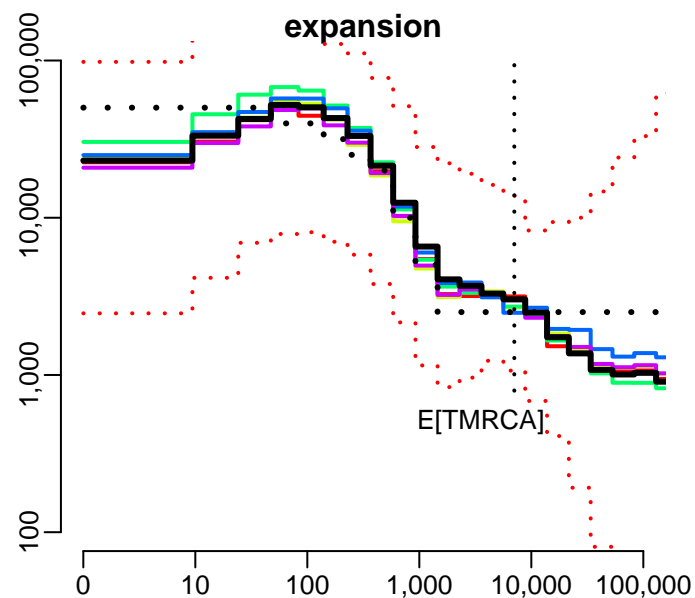**bottleneck1 old large**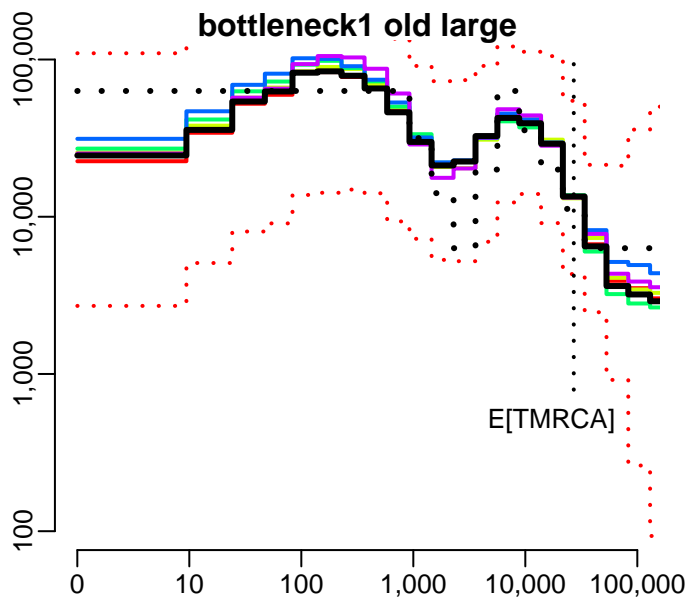**zigzag large**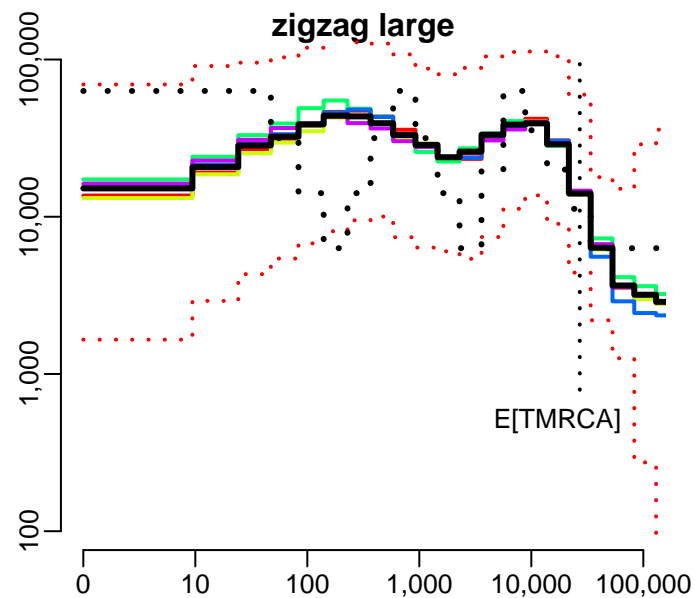

Supplement: S22 Fig — All settings are similar to Fig 3, except that AFS statistics were computed only from SNPs with a MAF above 20%. (PDF) [file pgen.1005877.s022.pdf]

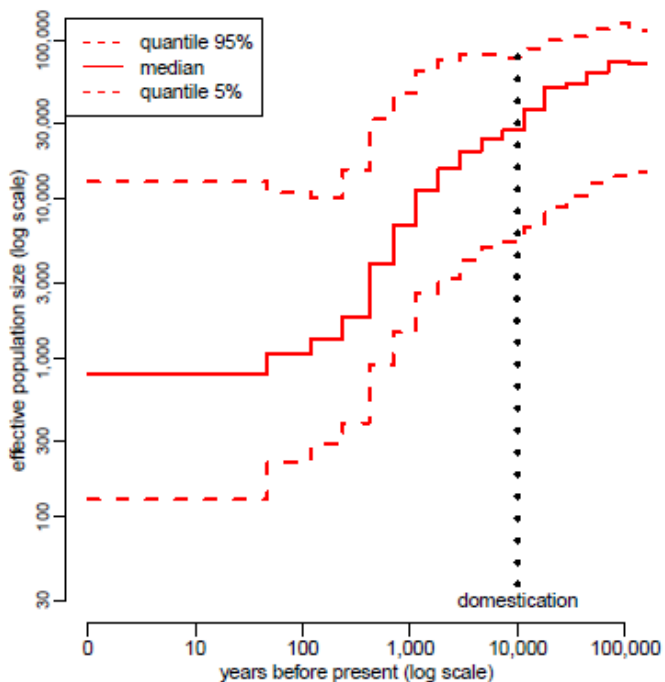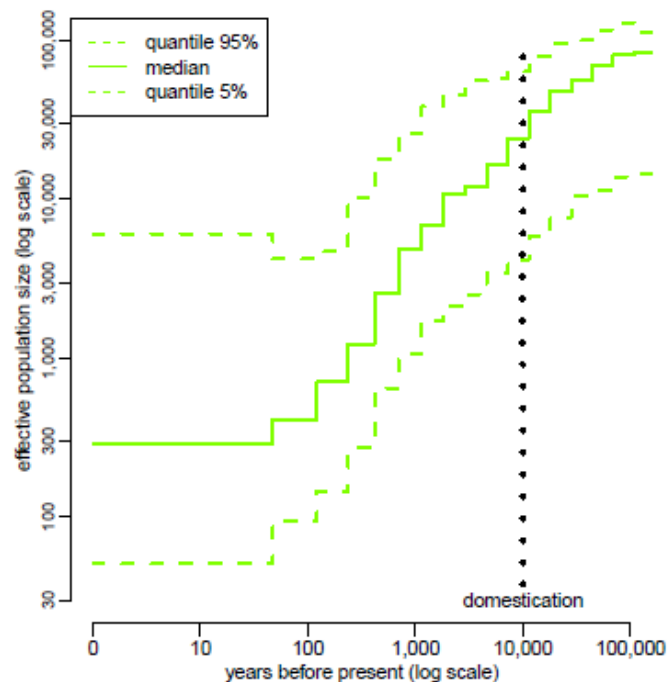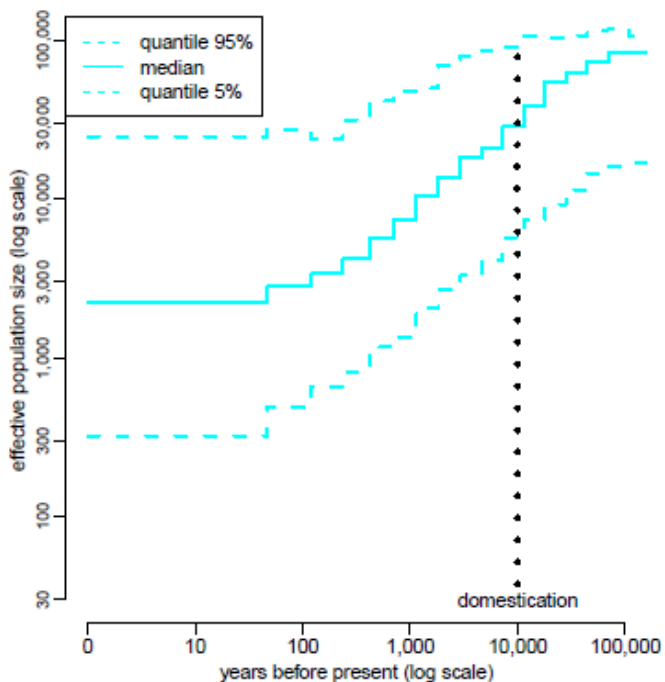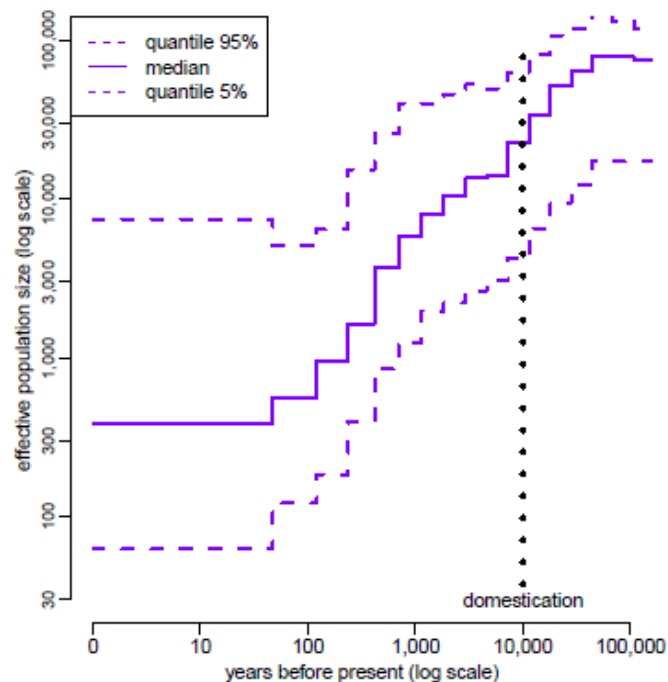

Supplement: S23 Fig — Holstein (top left), Angus (top right), Fleckvieh (bottom left) and Jersey (bottom right). Parameter settings are the same as in Fig 6. (PDF) [file pgen.1005877.s023.pdf]

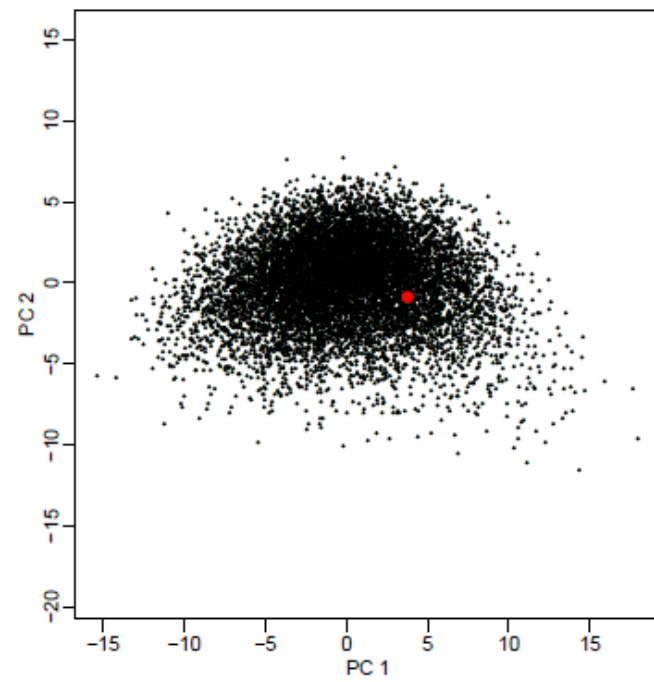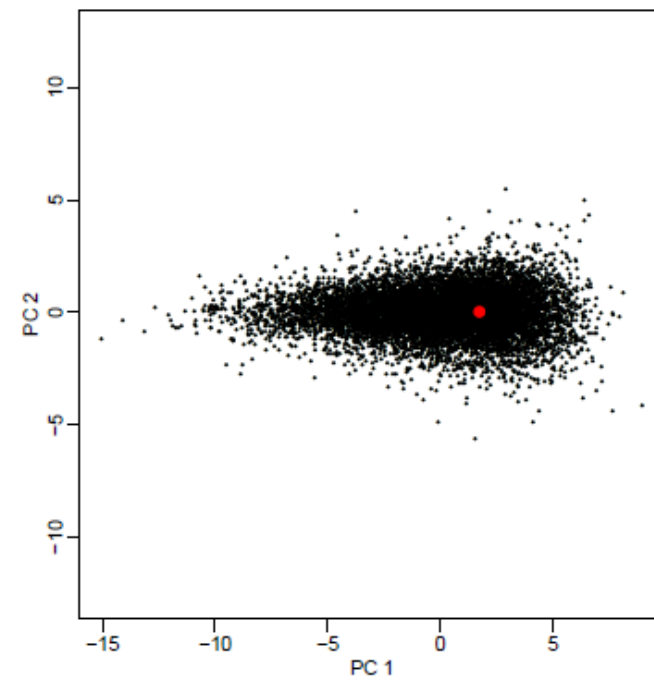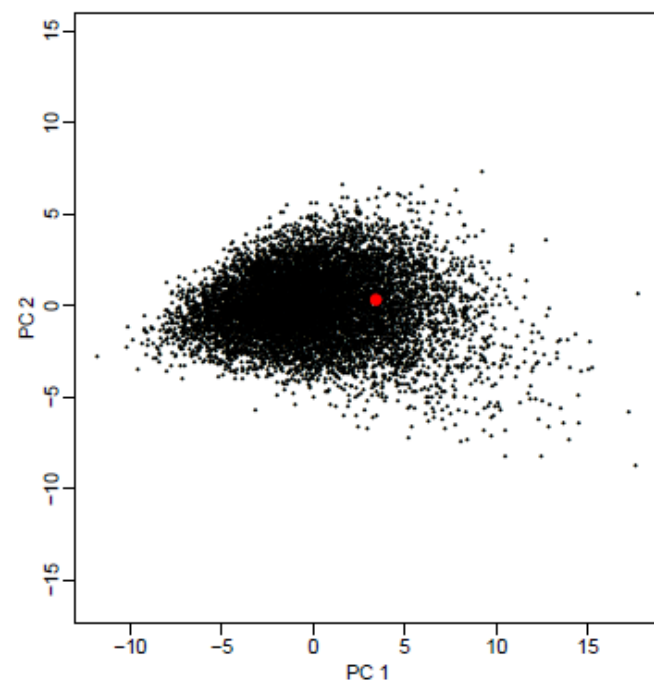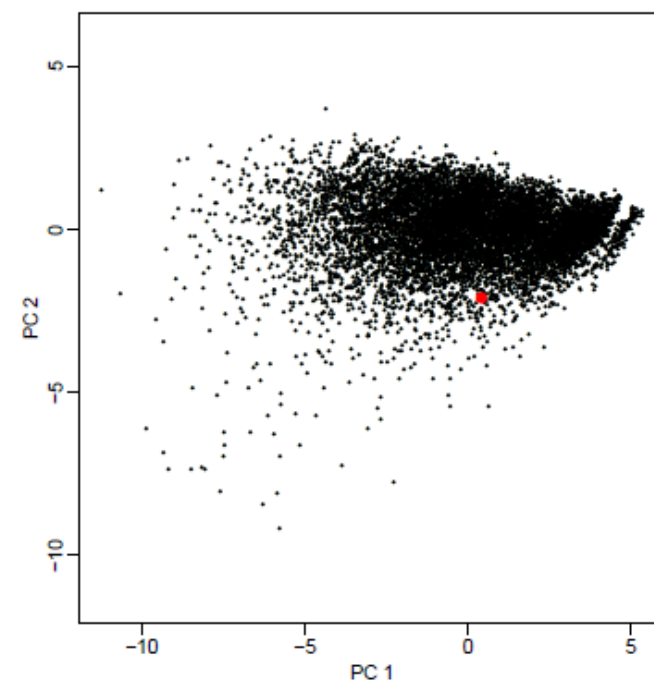

Supplement: S24 Fig — Ten thousand genomic samples were simulated under population size histories that were sampled from the posterior distribution estimated in Fig 6. Four combinations of summary statistics were computed from each sample: AFS and LD statistics (top left), AFS statistics alone (top right), LD statistics alone (bottom left) and IBS statistics (bottom right, see the Methods for a detailed description of these statistics). For each of these combinations, a principal component analysis (PCA) of the 10,000 simulated samples was performed: the projection of all samples on the two first dimensions of this PCA are plotted in black. The vector of summary statistics observed in Holstein was then projected on the same hyperplan. It always fell within the cloud of simulated summary statistics, which shows that the estimated history is able to reproduce summary statistics that are indeed similar to the observed ones. Interestingly, this also holds for IBS statistics, which were not used for the estimation. Results are shown for the Holstein breed but they were similar for the other breeds. (PDF) [file pgen.1005877.s024.pdf]

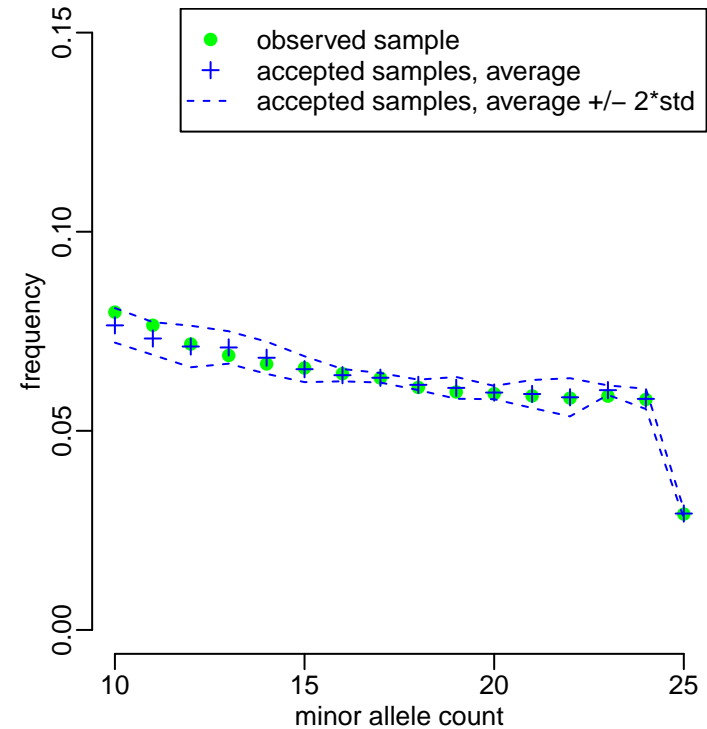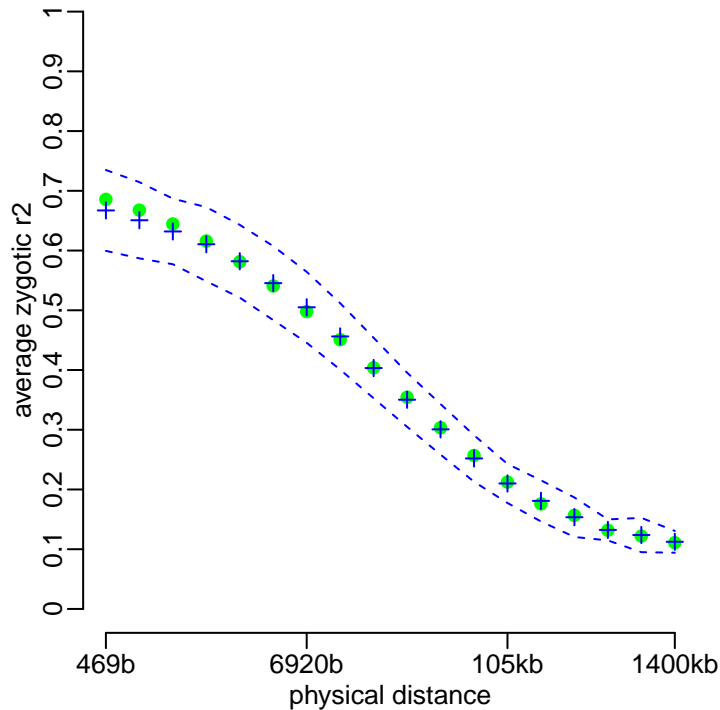

Supplement: S25 Fig — Observed AFS (left) and LD (right) statistics are shown by green full circles. The average value of these statistics over the five best simulated data sets, i.e. the five simulated data sets leading to the smallest distance between observed and simulated statistics, are shown by blue crosses. The variation of these statistics over the five best simulated data sets is also indicated by blue dotted lines, which correspond to the average value plus (or minus) twice the standard deviation of each statistic. (PDF) [file pgen.1005877.s025.pdf]

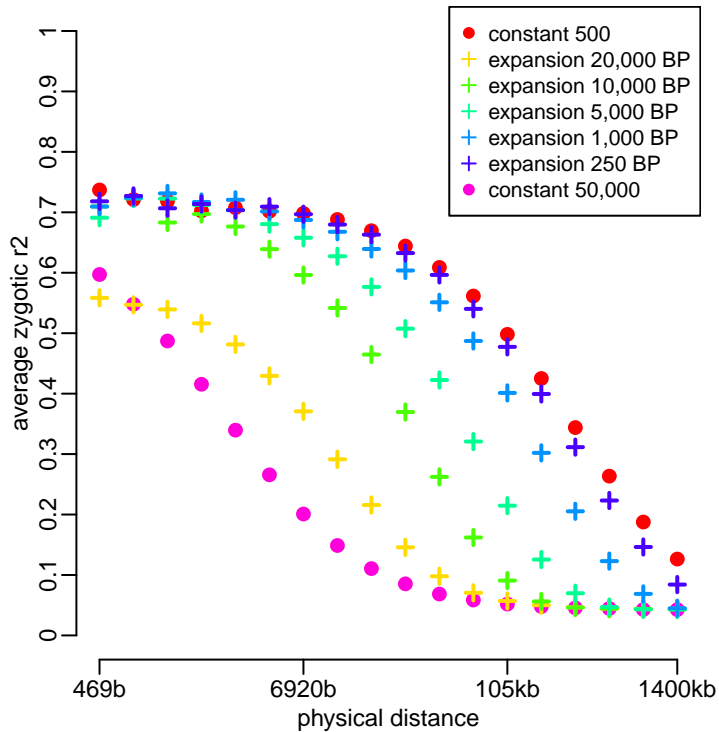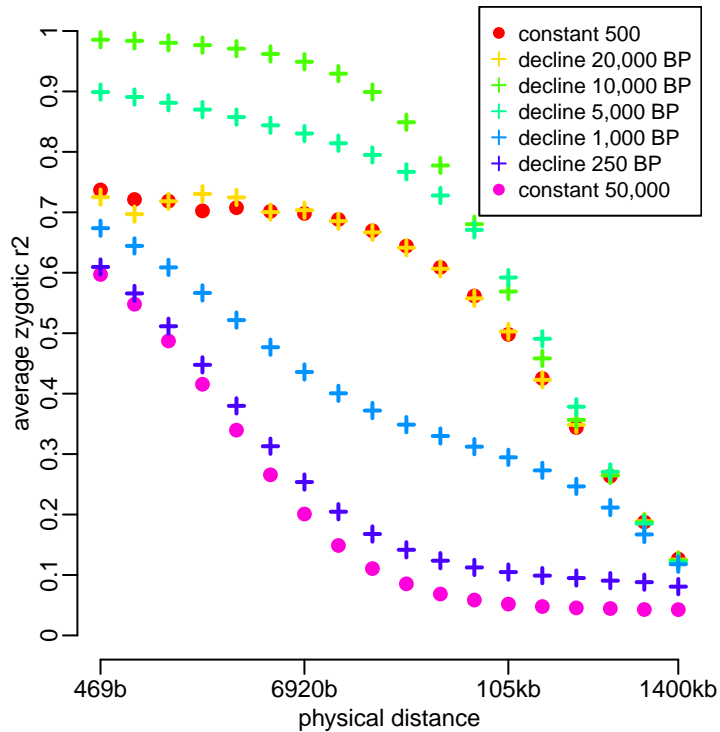

Supplement: S26 Fig — LD statistics for several scenarios inplying a sudden expansion from 500 to 50,000 individuals (left) or a sudden decline from 50,000 to 500 individuals (right). Several expansion or decline times were considered, as well as two scenarios with a constant population size of 500 or 50,000 individuals (see the legend). For each scenario, LD statistics were averaged over 20 PODs including 25 diploid genomes and 100 2Mb-long regions. In contrast with expansion scenarios, some decline scenarios lead to even larger LD statistics than those obtained for a constant small population. Indeed, as these declines are very old compared to the expected TMRCA of a population of 500 individuals, their main effect is to increase, at some loci, the time during which the sample has only two ancestral lineages. Because this increase is very large (backward in time, population size, and thus expected coalescence time, are suddenly multiplied by 100), mutations occuring in this part of the coalescence tree eventually represent a large proportion of all oberved polymorphic sites. Besides, for two linked loci with similar topologies of the coalescence tree, mutations occuring in this part of the tree lead to very high r2 values, up to 1 if the topologies are exactly the same. (PDF) [file pgen.1005877.s026.pdf]

small

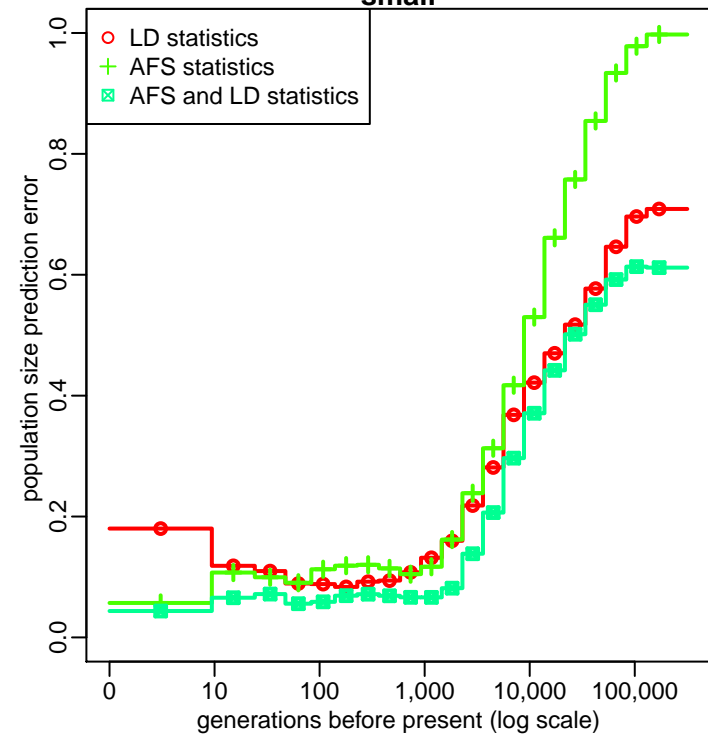

large

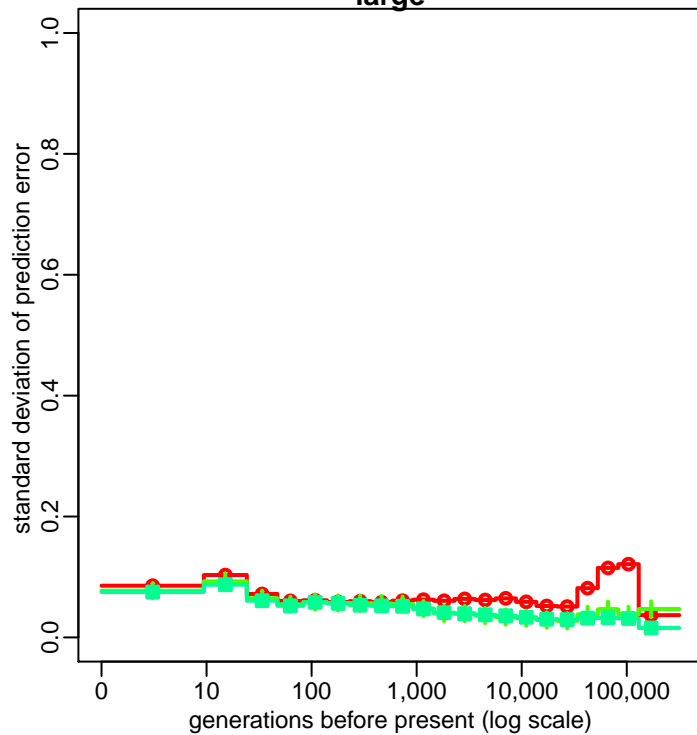

decline

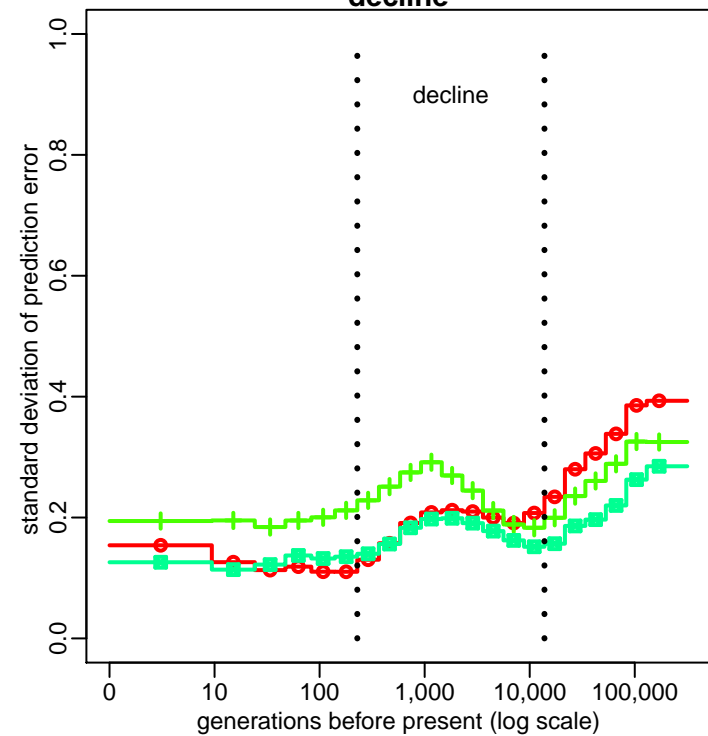

expansion

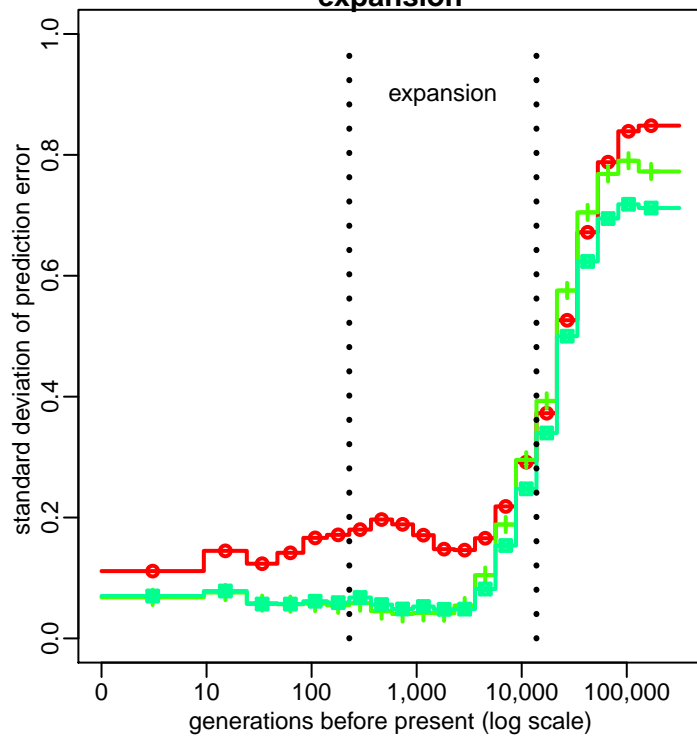

Supplement: S27 Fig — Prediction error for the estimated population size in each time window, focusing on scenarios with a population size below 1,000 (top left), above 10,000 (top right), below 1,000 in the last 200 generations and above 10,000 for times more ancient than 13,000 generations BP (bottom left) or above 10,000 in the last 200 generations and below 1,000 for times more ancient than 13,000 generations BP (bottom left). For the two latter scenarios, the time window where population size goes from above 10,000 to below 1,000 (or vice versa) is delimited by vertical dotted lines. For each scenario category, PE were evaluated from 2,000 random histories. Summary statistics considered in the ABC analysis were either the AFS statistics alone, the LD statistics alone or the AFS and LD statistics together (see the legend). All other settings are similar to Fig 2. (PDF) [file pgen.1005877.s027.pdf]

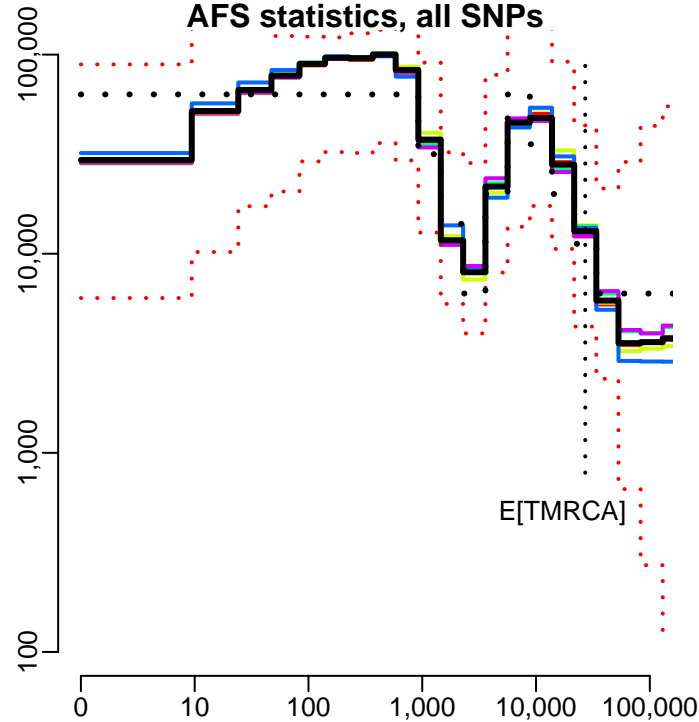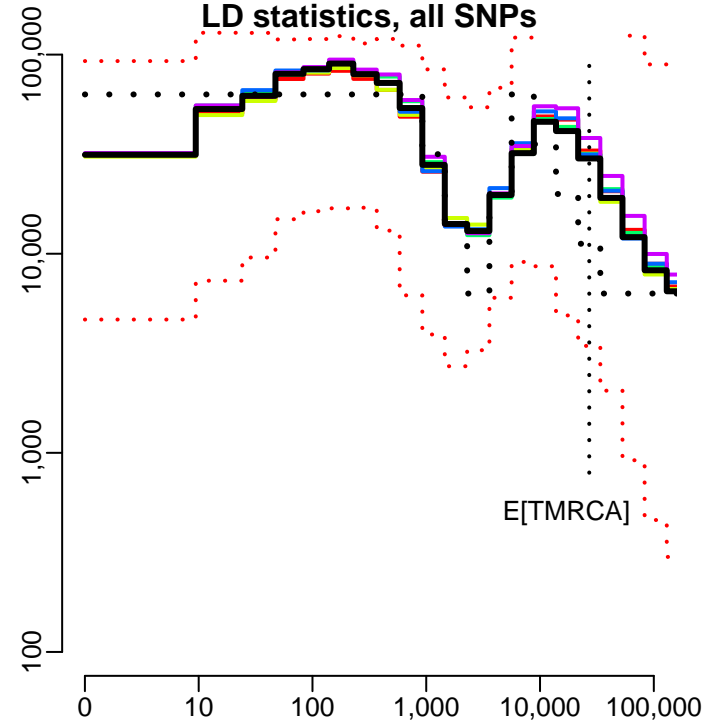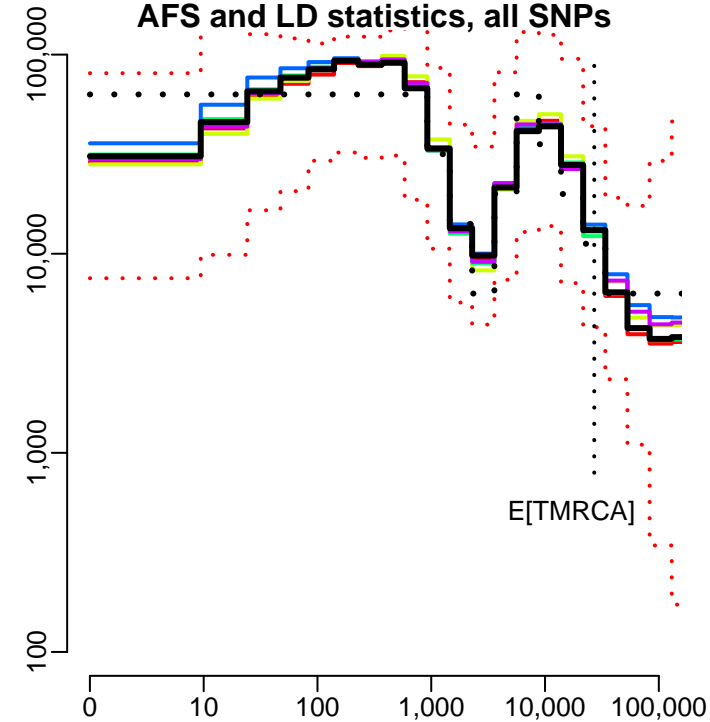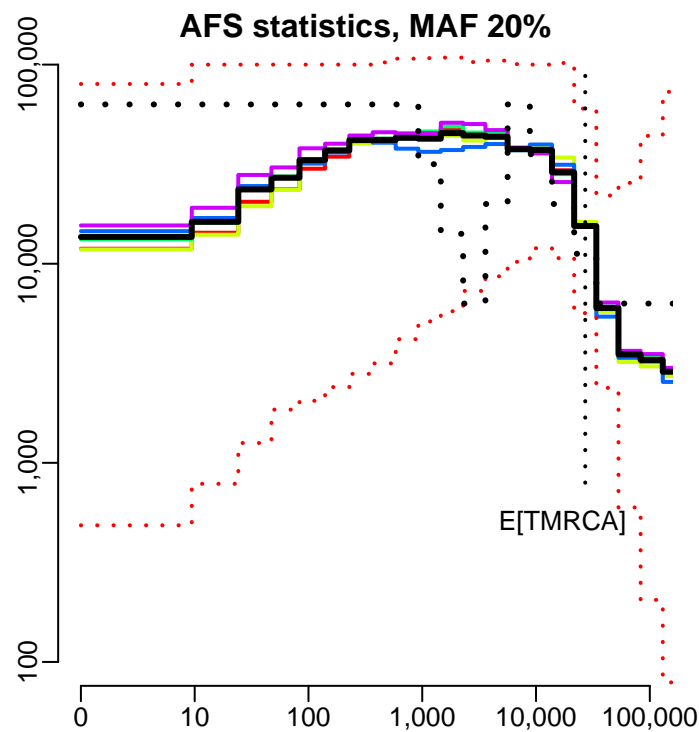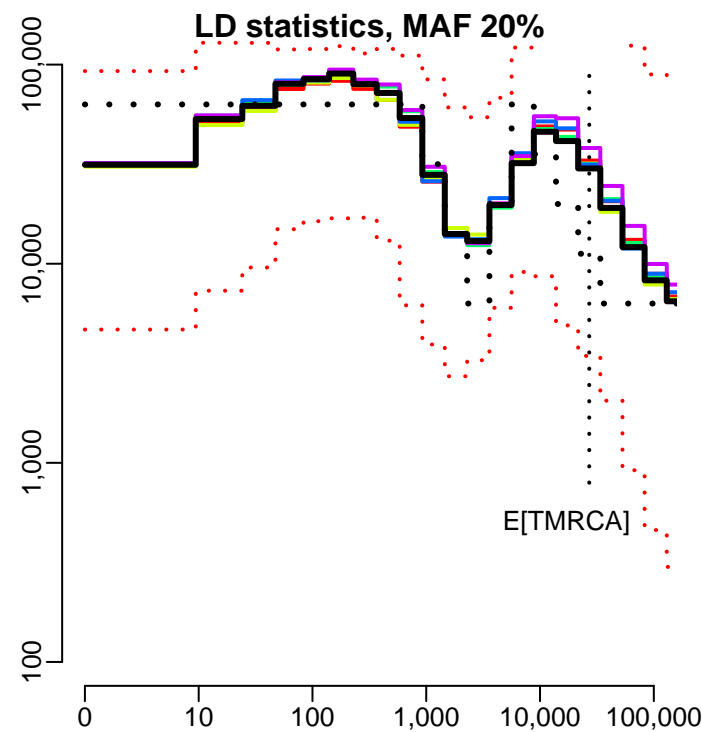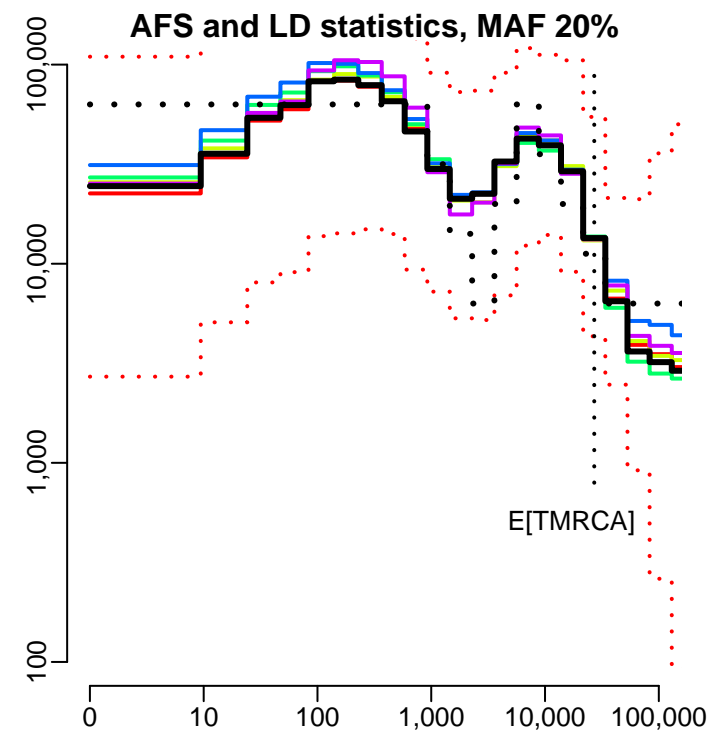

Supplement: S28 Fig — Summary statistics considered in the ABC analysis were either the AFS statistics alone (left column), the LD statistics alone (middle column), or the AFS and LD statistics together (right column). AFS statistics were computed using either all SNPs (top panels) or only those with a MAF above 20% (bottom panels). All other settings are similar to Fig 3. (PDF) [file pgen.1005877.s028.pdf]

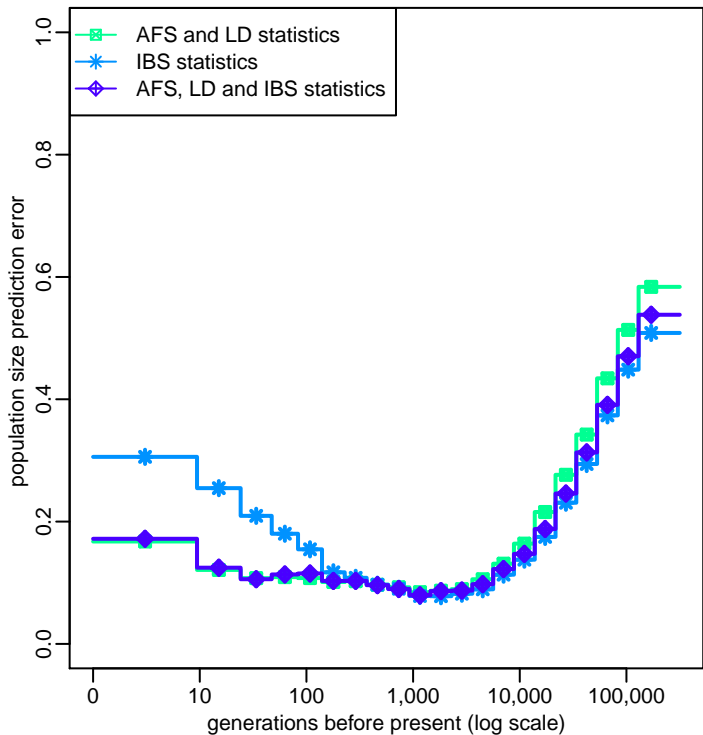

Supplement: S29 Fig — Prediction error for the population size in each time window, evaluated from 2,000 random population size histories. Summary statistics considered in the ABC analysis included several combinations of (i) the AFS, (ii) the average zygotic LD for several distance bins and (iii) the distribution of IBS segment lengths within one diploid individual. These statistics were computed from n = 25 diploid individuals, using all SNPs for AFS and IBS statistics and SNPs with a MAF above 20% for LD statistics. Other parameter settings are the same as in Fig 2. (PDF) [file pgen.1005877.s029.pdf]

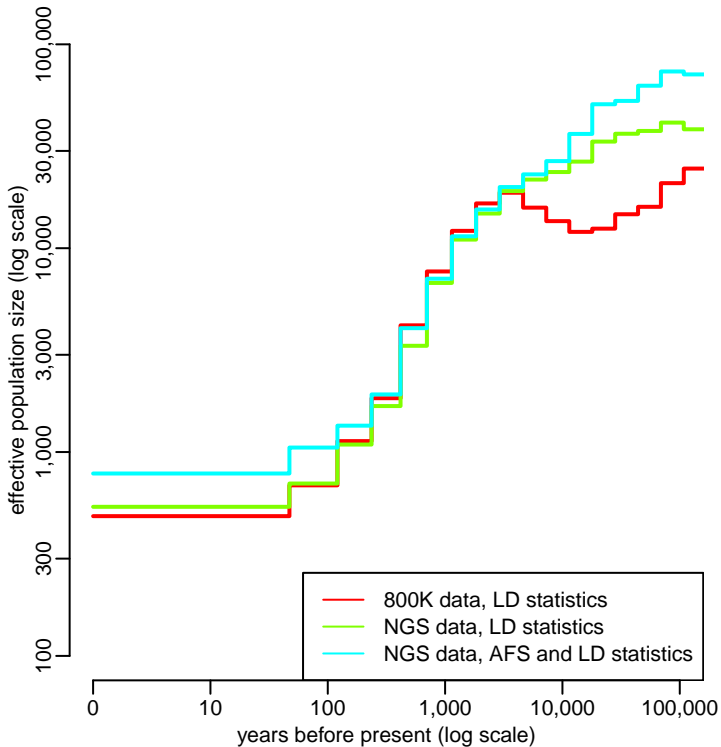

Supplement: S30 Fig — Estimation of population size history in the Holstein cattle breed using ABC, based on whole genome NGS data from n = 25 animals. Summary statistics considered in the ABC analysis included different combinations of (i) the AFS and (ii) the average zygotic LD for several distance bins. These statistics were computed either from the SNPs that are included in the 800K SNP chip or from all SNPs found in the NGS data. A MAF threshold of 20% was used for all curves and statistics. Other parameter settings are the same as in Fig 5. (PDF) [file pgen.1005877.s030.pdf]

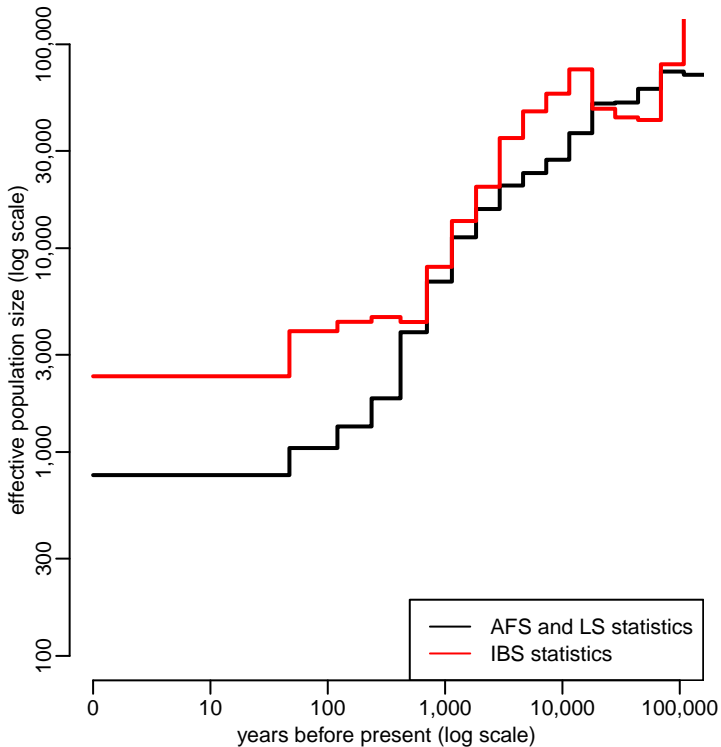

Supplement: S31 Fig — Estimation of population size history in the Holstein cattle breed using ABC, based on whole genome NGS data from n = 25 animals. Summary statistics considered in the ABC analysis were either both the AFS and the average zygotic LD for several distance bins, or the distribution of IBS segment lengths within one diploid individual. These statistics were computed using SNPs with a MAF above 20%. Other parameter settings are the same as in Fig 5. (PDF) [file pgen.1005877.s031.pdf]
