# Supplementary material for: Inferring Population Size History from Large Samples of Genome-Wide Molecular Data - An Approximate Bayesian Computation Approach
Source: PLoS Genet. 2016 Mar 4;12(3):e1005877. doi: 10.1371/journal.pgen.1005877 (PMC4778914; doi:10.1371/journal.pgen.1005877)
Supplement: S11 Fig — a scenario where all population sizes are divided by ten compared to the original zigzag (“zigzag small”, top right), a scenario where only the recent bottleneck of the original zigzag is simulated (“bottleneck1 recent large”, middle left), a scenario corresponding to the history wrongly inferred by ABC based on data from the “bottleneck1 recent large” scenario (middle right), and two scenarios where only the recent (bottom left) or the old (bottom right) bottleneck of the “zigzag small” are simulated. All settings are similar to Fig 3. (PDF) [file pgen.1005877.s011.pdf]

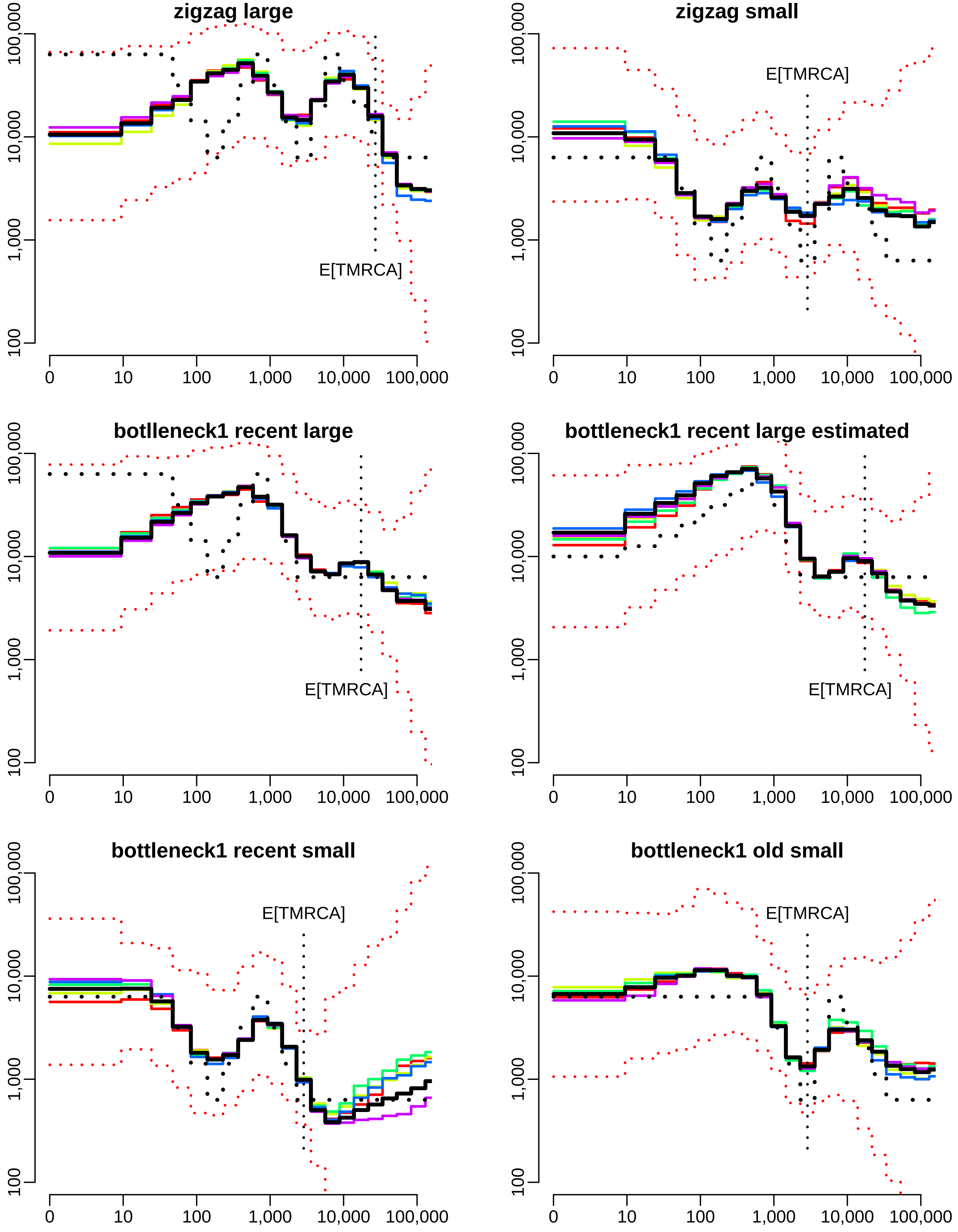
zigzag large
zigzag small
botlleneck1 recent large
bottleneck1 recent large estimated
bottleneck1 recent small
bottleneck1 old small
E[TMRCA]
100
1,000
10,000
100,000
0
10
100
1,000
10,000
100,000
