# Supplementary material for: Inferring Population Size History from Large Samples of Genome-Wide Molecular Data - An Approximate Bayesian Computation Approach
Source: PLoS Genet. 2016 Mar 4;12(3):e1005877. doi: 10.1371/journal.pgen.1005877 (PMC4778914; doi:10.1371/journal.pgen.1005877)
Supplement: S14 Fig — a sudden (rather than continuous) decline from 40,000 to 300 individuals occurring 200 generations BP (top right), a sudden decline from 40,000 to 300 individuals occurring 1,000 generations BP (middle left), the same sudden decline followed by an expansion to 5,000 individuals occurring 580 generations BP (middle right) or an expansion to 1,000 individuals occurring 140 generations BP (bottom left), and a scenario similar to the continuous decline (top left) but including a sudden decline to 100 individuals between 230 and 140 generations BP, followed by an expansion to 1,000 individuals (bottom right). All settings are similar to Fig 3. (PDF) [file pgen.1005877.s014.pdf]

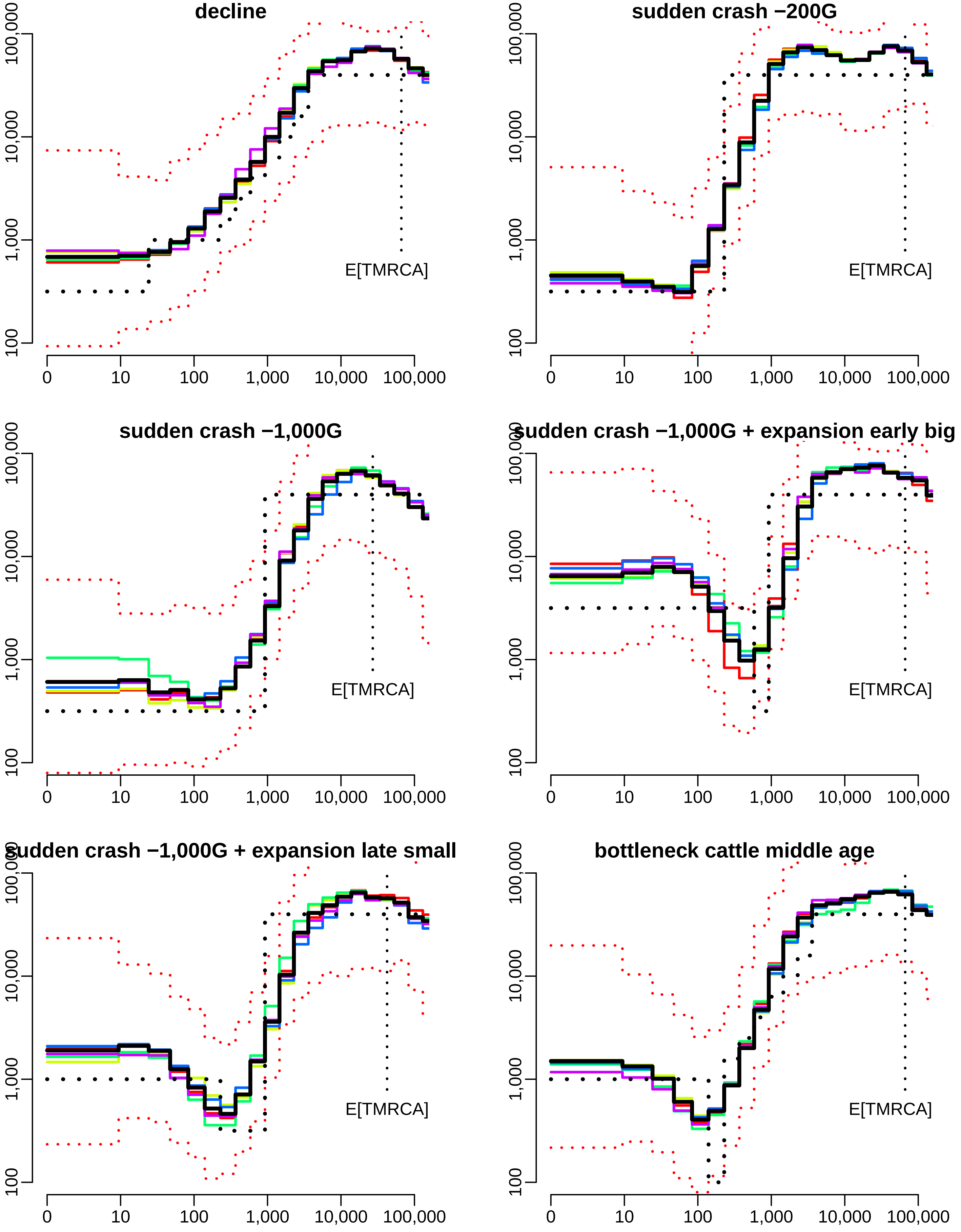

decline
sudden crash −200G
sudden crash −1,000G
sudden crash −1,000G + expansion early big
sudden crash −1,000G + expansion late small
bottleneck cattle middle age
E[TMRCA]
100
1,000
10,000
100,000
0
10
100
1,000
10,000
100,000
