# Supplementary material for: Inferring Population Size History from Large Samples of Genome-Wide Molecular Data - An Approximate Bayesian Computation Approach
Source: PLoS Genet. 2016 Mar 4;12(3):e1005877. doi: 10.1371/journal.pgen.1005877 (PMC4778914; doi:10.1371/journal.pgen.1005877)
Supplement: S17 Fig — For each scenario, the five PODs considered for MSMC estimation were the same as in S14 Fig. The expected TMRCA shown here is also the same as in S14 Fig, it corresponds to samples of 50 haploid sequences. (PDF) [file pgen.1005877.s017.pdf]

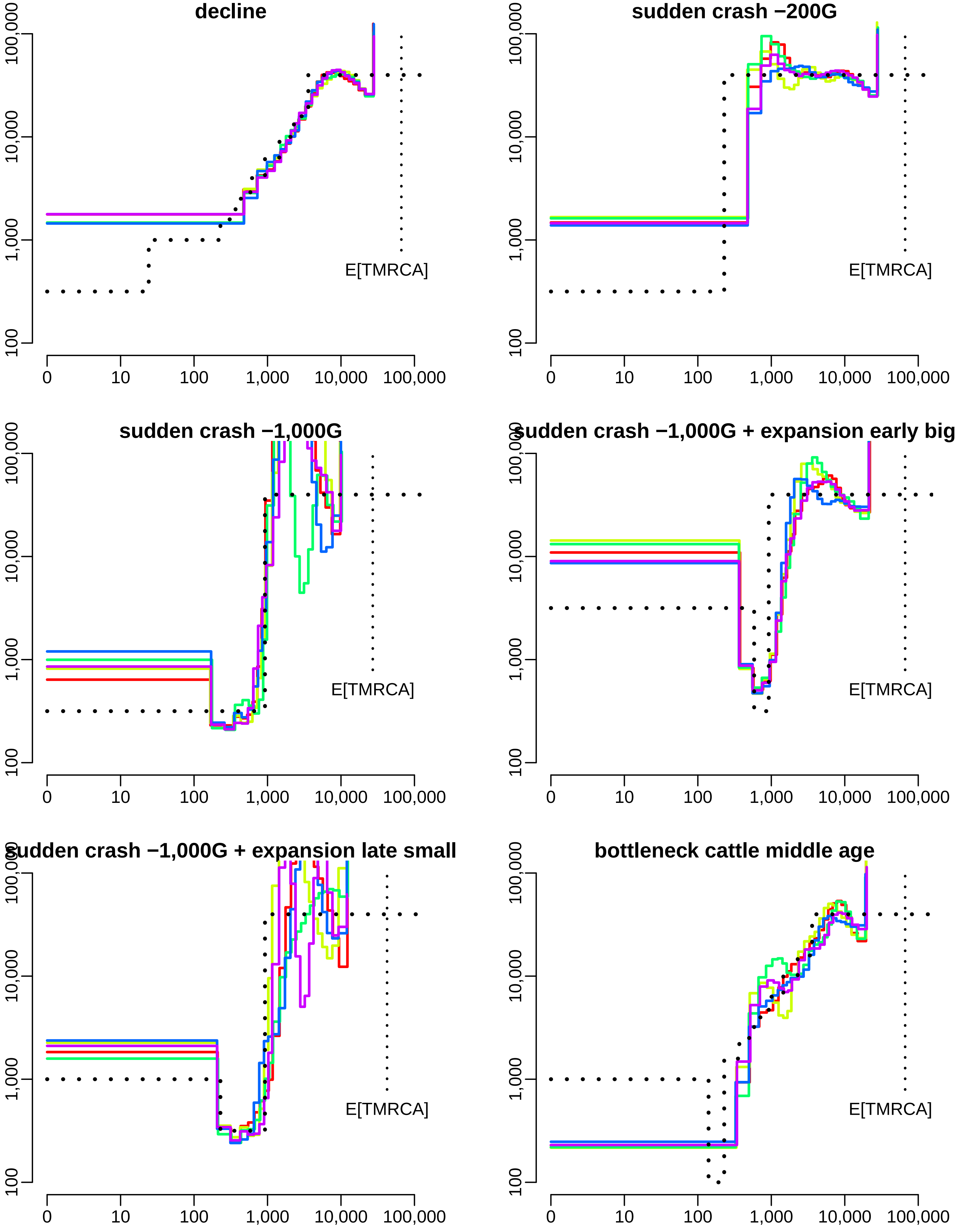

decline
sudden crash −200G
sudden crash −1,000G
sudden crash −1,000G + expansion early big
sudden crash −1,000G + expansion late small
bottleneck cattle middle age
100
1,000
10,000
100,000
0
10
100
1,000
10,000
100,000
E[TMRCA]
