# Supplementary material for: Inferring Population Size History from Large Samples of Genome-Wide Molecular Data - An Approximate Bayesian Computation Approach
Source: PLoS Genet. 2016 Mar 4;12(3):e1005877. doi: 10.1371/journal.pgen.1005877 (PMC4778914; doi:10.1371/journal.pgen.1005877)
Supplement: S19 Fig — Estimation of past effective population size using MSMC with four haplotypes in the “small” scenario (top), the “decline” scenario (middle) and the “expansion” scenario (bottom). MSMC analyzes were run from perfectly phased data, phased data with 1 or 10 switch errors per Mb and diploid individual, or unphased data (i.e. two unphased diploid individuals). All other settings are similar to S15 Fig. (PDF) [file pgen.1005877.s019.pdf]

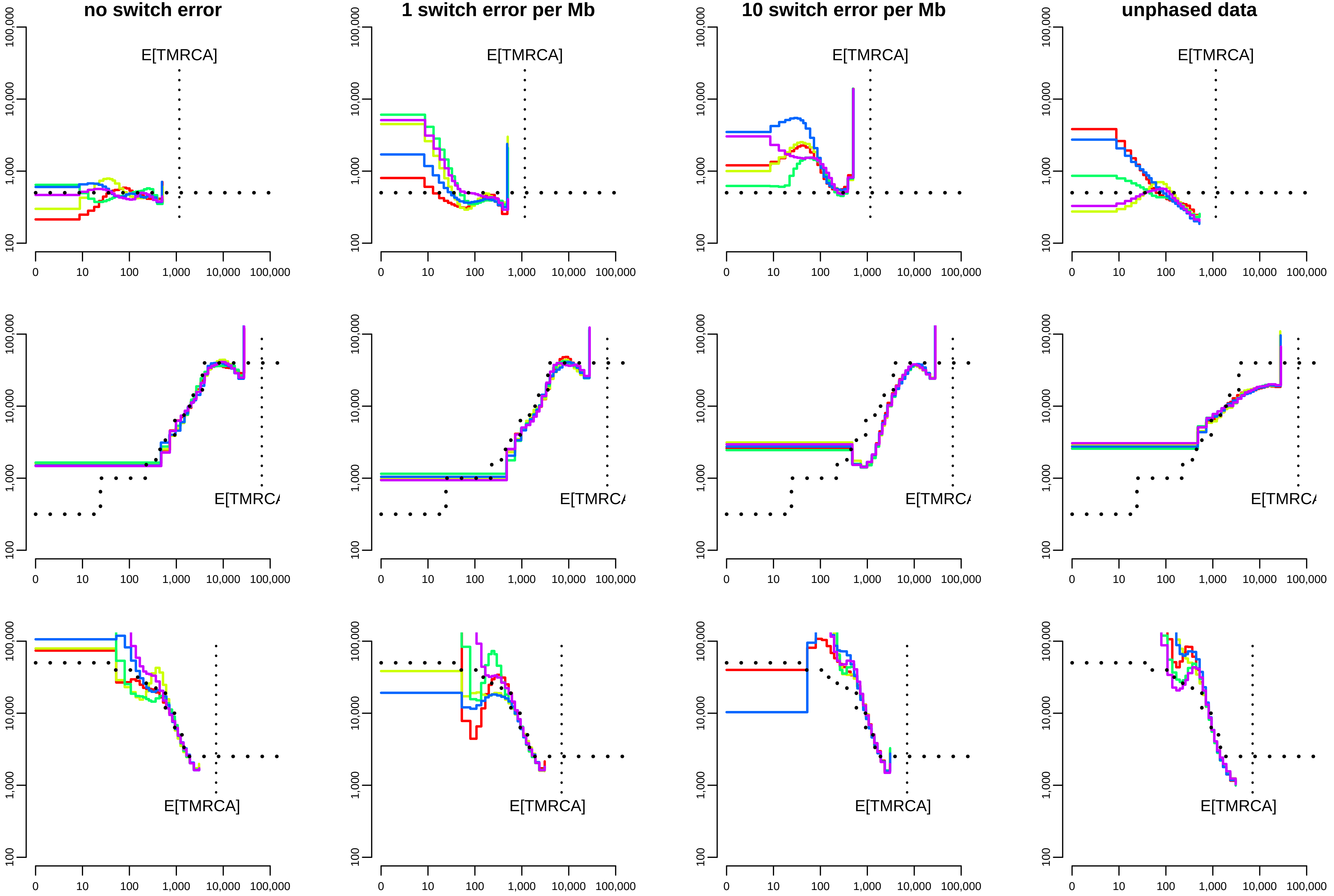

no switch error
1 switch error per Mb
10 switch error per Mb
unphased data
E[TMRCA]
100
1,000
10,000
100,000
0
10
100
1,000
10,000
100,000
